# Multinucleated Giant Cells Are Specialized for Complement-Mediated Phagocytosis and Large Target Destruction

**DOI:** 10.1016/j.celrep.2015.10.065

**Published:** 2015-11-25

**Authors:** Ronny Milde, Julia Ritter, Glenys A. Tennent, Andrzej Loesch, Fernando O. Martinez, Siamon Gordon, Mark B. Pepys, Admar Verschoor, Laura Helming

**Affiliations:** 1 Institute for Medical Microbiology, Immunology and Hygiene, Technische Universität München, 81675 Munich, Germany; 2 Wolfson Drug Discovery Unit, Centre for Amyloidosis and Acute Phase Proteins, Division of Medicine, Royal Free Campus, University College London, London NW3 2PF, UK; 3 Kennedy Rheumatology Institute, University of Oxford, Oxford OX3 7LD, UK; 4 Sir William Dunn School of Pathology, University of Oxford, Oxford OX1 3RE, UK; 5 Institute for Systemic Inflammation Research, Universität zu Lübeck, 23538 Lübeck, Germany

## Abstract

Multinucleated giant cells (MGCs) form by fusion of macrophages and are presumed to contribute to the removal of debris from tissues. In a systematic in vitro analysis, we show that IL-4-induced MGCs phagocytosed large and complement-opsonized materials more effectively than their unfused M2 macrophage precursors. MGC expression of complement receptor 4 (CR4) was increased, but it functioned primarily as an adhesion integrin. In contrast, although expression of CR3 was not increased, it became functionally activated during fusion and was located on the extensive membrane ruffles created by excess plasma membrane arising from macrophage fusion. The combination of increased membrane area and activated CR3 specifically equips MGCs to engulf large complement-coated targets. Moreover, we demonstrate these features in vivo in the recently described complement-dependent therapeutic elimination of systemic amyloid deposits by MGCs. MGCs are evidently more than the sum of their macrophage parts.

## Highlights

• MGCs are specialized for phagocytosis of large and complement-opsonized particles
• MGCs show extensive membrane ruffles containing pre-activated complement receptor 3
• Membrane ruffles provide excess membrane for ingestion of large materials
• MGCs eliminate systemic amyloid deposits after immunotherapeutic targeting

## Graphical Abstract

**Figure:**
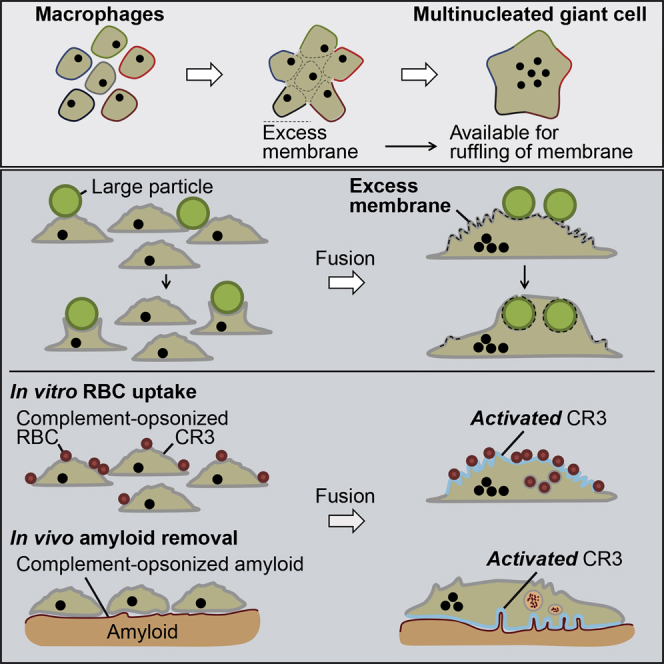

## Teaser

Macrophage-derived multinucleated giant cells (MGCs) form in diverse chronic inflammatory diseases, but their functional role remains unclear. Milde et al. show that MGCs are specialized for complement-mediated phagocytosis and destruction of large targets and demonstrate their key role in the therapeutic elimination of the pathogenic amyloid deposits in systemic amyloidosis.

## Introduction

Multinucleated giant cells (MGCs), first described in tuberculosis (Langhans, 1868), are also present in diverse infectious and non-infectious chronic inflammatory conditions, including schistosomiasis, atherosclerosis, sarcoidosis, and Langerhans cell histiocytosis (Helming and Gordon, 2009, Samokhin et al., 2010). MGCs also typify the foreign body reaction to macroscopic organic and inorganic materials, such as uric acid crystals and surgical implants (Helming and Gordon, 2009, Lai and Zhou, 2013). MGCs and osteoclasts are derived by cell-cell fusion of macrophages. Formation of osteoclasts, essential for bone resorption, is mediated by receptor activator of nuclear factor kappa-B ligand (RANKL) and macrophage colony-stimulating factor (M-CSF). Factors inducing MGC formation are less well defined (Helming and Gordon, 2009), but interleukin-4 (IL-4), a T_H_2 cytokine of alternative (M2) macrophage activation, induces fusion in vitro and in sarcoidosis and foreign body reactions in vivo (Kao et al., 1995, Prokop et al., 2011). The role of MGCs in disease is also obscure, and it remains unclear whether they are beneficial or detrimental to disease outcome. It cannot be excluded that fused macrophages exhibit different roles depending on the nature of the disease. As they are often found under conditions where large and/or poorly degradable material is present (e.g., implants and uric acid crystals), there is speculation about specialization of MGCs for uptake of large particles (Anderson et al., 2008), but there are no rigorous quantitative studies. Indeed, reduced (Chambers, 1977, Lay et al., 2007), increased (Moreno et al., 2007, Nakanishi-Matsui et al., 2012), or unchanged (Schlesinger et al., 1984) phagocytic activity of MGCs compared to non-fused macrophages have all been reported. However, all of these studies lacked unambiguous discrimination between fully ingested particles and those loosely attached to the external cell surface. Here, we report a direct and well-controlled systematic comparison of the phagocytic activity of MGCs and M2 macrophages in vitro and characterize the cellular mechanisms underlying the unique functional behavior of MGCs.

Furthermore, we demonstrate these features in vivo in the recently described complement-dependent therapeutic elimination of systemic amyloid deposits by MGCs. This process is characterized by antibody-mediated complement activation and opsonization of amyloid deposits, triggering macrophage infiltration and formation of MGCs, which efficiently eliminate the amyloid (Bodin et al., 2010, Richards et al., 2015). We show here that this therapeutic process involves the same phenotypic features of MGCs that characterize them in vitro.

## Results

### MGCs Exhibit Enhanced Phagocytic Activity toward Complement-Opsonized Targets

**Figure 1:**
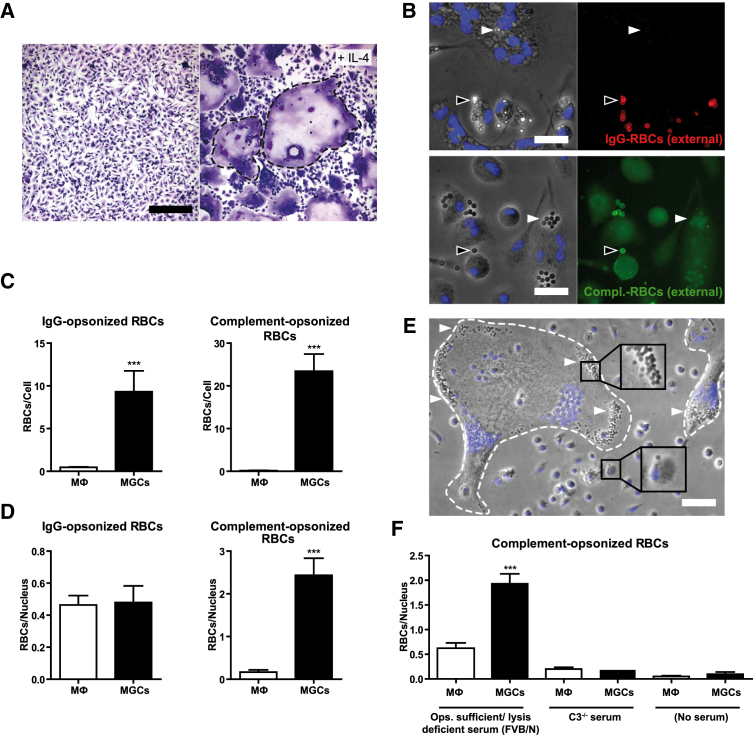
Phagocytosis of Complement-Opsonized RBCs by MGCs Is Increased when Compared to Unfused M2 Macrophages (A) Macrophage fusion induced by IL-4 treatment of BMMs, Hemacolor staining, observed in bright field microscopy. Dashed lines indicate MGCs. The scale bar represents 200 μm. (B) Discrimination of external and internalized phagocytic targets. External IgG-opsonized RBCs were immunostained with anti-rabbit DyLight549 antibodies (red) and external complement-opsonized RBCs with anti-iC3b and Alexa-488-labeled secondary antibodies (green). Arrows indicate non-ingested (black) or ingested (white) particles. Nuclei are counterstained with Hoechst (blue). Results were viewed in fluorescence microscopy. The scale bar represents 20 μm. (C) Quantification of internalized particles in unfused macrophages and MGCs after 1 hr of phagocytosis, particle per cell ratio. (D) Quantification of internalized particles, particle per nucleus ratio. (E) Phase contrast image overlaid with nuclear staining (Hoechst; blue) after incubation with complement-opsonized RBCs. RBCs (arrows) are mainly detected in MGCs (dashed lines). The scale bar represents 50 μm. (F) Involvement of C3 in phagocytosis of serum-opsonized RBCs. RBCs were left unopsonized or opsonized with FVB/N (C5-deficient) serum or serum from C3-KO mice and added to the MGC/macrophage cultures for 1 hr. Internalized RBCs were quantified and normalized to the number of nuclei (C, D, and F) and shown as mean ± SEM; n = 25. Results are representative of greater than or equal to three independent experiments. Statistical comparison was with macrophages under the same conditions. ^∗∗∗^p < 0.001; two-tailed Student’s t test.

Fusion of murine primary bone marrow-derived macrophages (BMMs) was induced by IL-4 (Figure 1A), resembling M2 macrophage activation, and the phagocytic capacities of fused and non-fused macrophages were evaluated with sheep red blood cells (RBCs) opsonized either with IgG anti-RBC antibody alone or with IgM anti-RBC antibody followed by fresh whole C5-deficient mouse serum to provide complement. Specific fluorescent-labeled antibodies directed against the opsonizing agent (Figure 1B) were used to discriminate between bound and internalized particles. Significantly more RBCs were internalized per multinucleated cell than per non-fused mononucleated M2-activated macrophage of the same culture, both for complement and IgG opsonins (Figure 1C). However, when the numbers of internalized RBCs were normalized to the number of fused macrophages per MGC, determined by the number of nuclei present, these remained much higher only for serum-opsonized particles (Figures 1D and 1E). For IgG-opsonized RBCs, the particle/nucleus ratio was comparable between MGCs and unfused M2 macrophages (Figure 1D). When C3-deficient serum was used for opsonization, phagocytosis was comparable to that of non-opsonized RBCs, confirming that C3 fixation was essential for enhanced uptake (Figure 1F).

### MGCs Are Specialized for Phagocytosis of Large Particles

**Figure 2:**
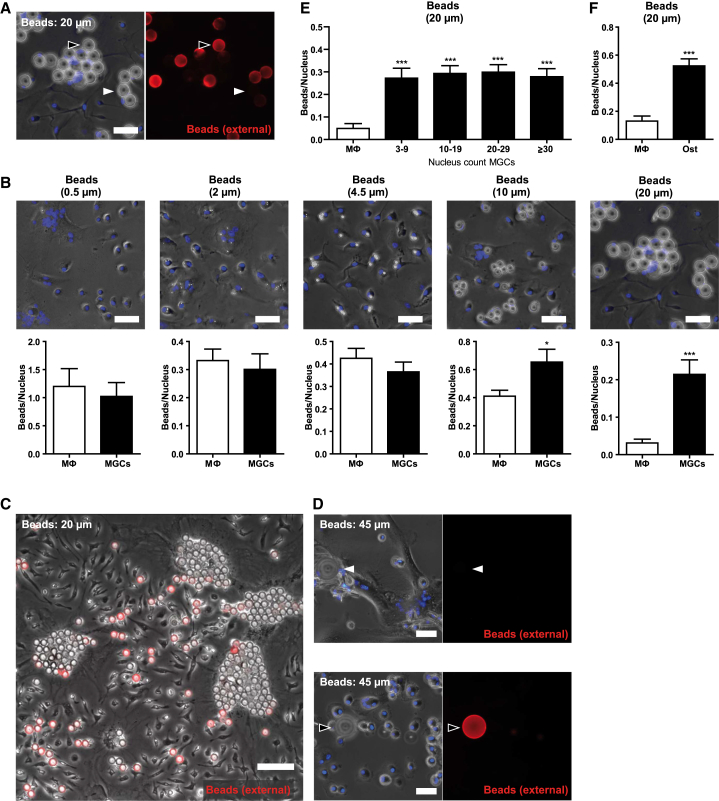
MGCs Are Specialized for the Phagocytosis of Large Particles (A) Detection of external rabbit IgG-coated latex beads. External beads were immunostained with anti-rabbit DyLight549 antibodies (red), nuclei were counterstained with Hoechst (blue), and results viewed by fluorescence microscopy. Arrows indicate non-ingested (black) or ingested (white) particles. The scale bar represents 30 μm. (B) Quantification of internalized beads of different sizes in macrophages and MGCs after 1 hr or 24 hr (20-μm beads) exposure, normalized to the nucleus count (n = 25). To illustrate absolute sizes of the different types of beads, phase contrast images overlaid with nuclear staining (Hoechst; blue) are shown. For quantification of internalized 0.5-μm beads, cells were permeabilized after staining and fixation and beads (re-)stained with Alexa-488-labeled anti-rabbit antibodies. The scale bar represents 30 μm. (C) Phase contrast image overlaid with immunostaining of external beads (DyLight549-labeled anti-rabbit antibodies; red) after incubation with 20-μm IgG beads. Beads were concentrated within MGCs. The scale bar represents 100 μm. (D) Detection of internalized 45-μm IgG-coated beads. Staining was performed as described in (A). Arrows indicate non-ingested (black) or ingested (white) particles. The scale bar represents 40 μm. (E) Bead uptake in MGCs grouped according to their nucleus count. Internalized beads were quantified in MGCs with 3–9, 10–19, 20–29, and ≥30 nuclei (n = 20). (F) Uptake of 20-μm IgG-coated latex beads in osteoclasts (Ost) and macrophages within the same culture, beads per nucleus ratio (n = 14). Shown are means ± SEM. Results are representative of greater than or equal to three independent experiments. ^∗^p < 0.05; ^∗∗∗^p < 0.001; two-tailed Student’s t test. See also Figure S1 and Movies S1 and S2.

The phagocytic capacity of fused and unfused M2 macrophages for non-organic particles of different sizes was compared using defined diameter, 0.5, 2, 4.5, 10, and 20 μm, polystyrene beads, with immunostaining to exclude non-internalized particles (Figure 2A). Normalizing the number of phagocytosed beads to the number of nuclei per MGC, beads of up to 4.5 μm were phagocytosed at comparable rates by M2-activated mono- and multinucleated macrophages (Figure 2B). In contrast, beads ≥10 μm were preferentially taken up by MGCs in mixed cultures (Figures 2B and 2C), even though macrophages have been reported to be able to ingest 20-μm beads (Cannon and Swanson, 1992). Beads of 45 μm diameter are arguably too large to be phagocytosed by individual macrophages, and their uptake was detected only in MGCs (Figure 2D), with complete ingestion confirmed by 3D visualization of confocal microscopy images (Figures S1A and S1B; Movies S1 and S2). The MGCs were evidently specialized for phagocytosis of large targets, but this was not simply a function of the larger cell size: with 20-μm beads, there was no significant difference in the internalized bead/nucleus ratio between MGCs with 3–9, 10–19, 20–29, and ≥30 nuclei (Figure 2E), demonstrating that phagocytosis of these large particles by MGCs was independent of cell size. Internalization of large particles is therefore an inherent trait of MGCs, distinctly different from their mononuclear M2 macrophage precursors.

In the aforementioned studies, beads that were covalently coated with nonspecific IgGs were used to aid their extra/intracellular detection. Beads with nonspecific, chemically bound IgG are internalized independently of Fc receptors (Michl et al., 1983). Uptake by MGCs of 10- and 20-μm beads coated with the irrelevant protein ovalbumin was equally increased, confirming that ingestion of these large beads was not dependent on Fc receptors (Figure S1C). Furthermore, bead internalization by MGCs was completely blocked by inhibition of actin polymerization and PI3 kinase, confirming a bona fide phagocytosis process by MGCs (Figure S1D). Larger 20- and 45-μm beads were phagocytosed more slowly than smaller particles, but, although incubation times had to be extended from 1 to 24 hr, the macrophage fusion index did not differ before and after incubation, confirming that internalization was indeed due to phagocytosis and not to fusion of macrophages around the large beads (Figure S1E). Interestingly, osteoclasts generated by treatment of macrophages with RANKL and M-CSF also efficiently phagocytosed 20-μm beads (Figure 2F), demonstrating that fusion of macrophages into multinucleated cells, mediated by different stimuli, leads to a generally enhanced ability to phagocytose large targets.

### Inflammatory Conditions Stimulate IgG-Mediated Phagocytosis by Macrophages, but Not MGCs

**Figure 3:**
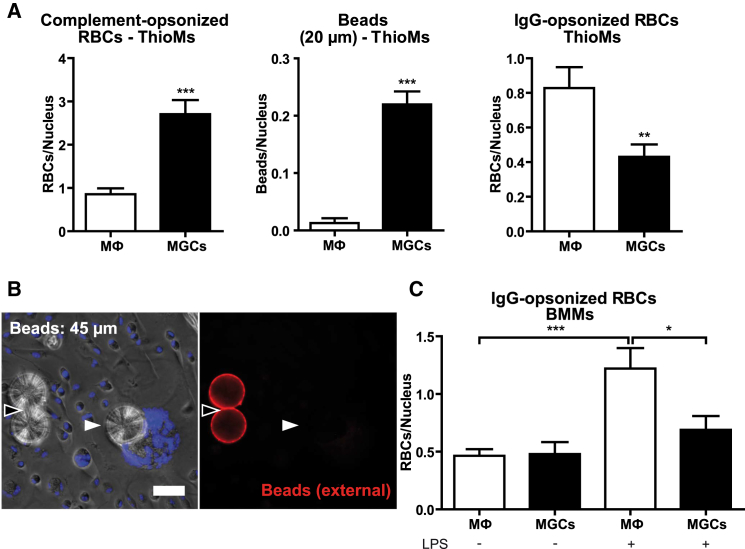
MGCs Show a Reduced Uptake of IgG-Opsonized RBCs Under Inflammatory Conditions (A) Quantification of internalized particles in thioglycollate-elicited peritoneal macrophages (ThioMs) and ThioM-derived MGCs, normalized to the number of nuclei. (B) Detection of internalized 45-μm IgG-coated latex beads. External beads were immunostained with anti-rabbit IgG DyLight549 antibodies (red), nuclei were counterstained with Hoechst (blue), and results were viewed by fluorescence microscopy. Internalized particles (white arrow) appear unstained and external (black arrow) in red. (C) Internalized IgG-opsonized RBCs in untreated and cells pretreated with LPS (10 ng/ml) for 24 hr, normalized to the number of nuclei. Results in (A) and (C) are shown as mean ± SEM, n = 25, and are representative of greater than or equal to three independent experiments. ^∗^p < 0.05; ^∗∗^p < 0.01; ^∗∗∗^p < 0.001; two-tailed Student’s t test.

The BMMs used to produce MGCs in vitro are resting macrophages, but in vivo MGCs generally form under chronic inflammatory conditions (Helming and Gordon, 2009). Thioglycollate-elicited inflammatory macrophages (ThioMs) were therefore tested and, as with BMM-MGCs (Figures 1 and 2), ThioM-derived IL-4-induced MGCs showed a clearly increased uptake of complement-opsonized RBCs (Figure 3A) as well as large polystyrene beads (Figures 3A and 3B). In contrast, ThioM-MGCs showed reduced phagocytosis of IgG-opsonized particles (Figure 3A). When IgG-opsonized particles were offered to BMMs and BMM-MGCs after exposure to LPS as an inflammatory stimulus in vitro, there was increased uptake by unfused macrophages, but not by MGCs (Figure 3C). Inflammatory conditions thus did not affect increased phagocytosis of complement-opsonized and large objects by MGCs, and IgG-mediated phagocytosis was increased only in macrophages.

### Physical Separation of MGCs from Unfused Macrophages Enables Direct Comparison of Phagocytic Receptor Expression

**Figure 4:**
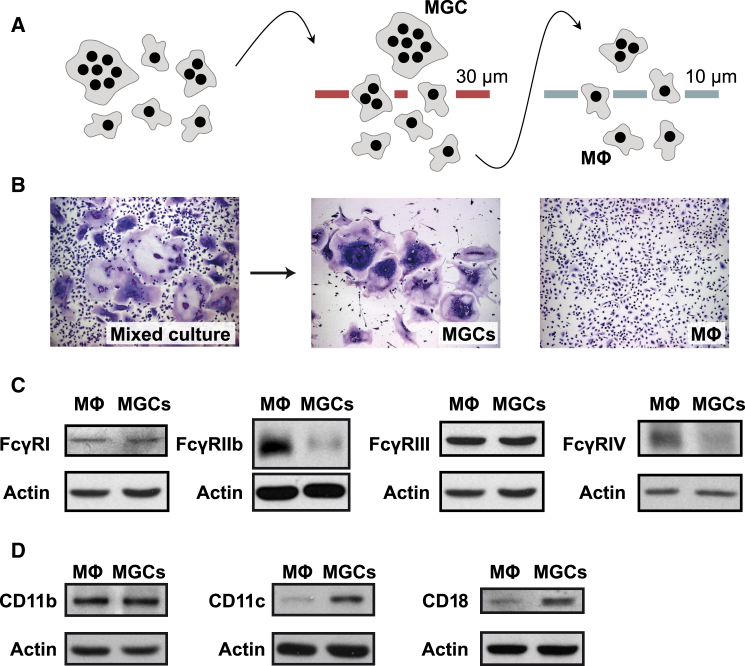
Altered Phagocytic Activity of MGCs Toward IgG- and C3-Opsonized RBCs Correlates with Protein Expression of the Respective Phagocytic Receptors (A) Experimental approach for separation of macrophages and MGCs from a mixed culture. BMMs were induced to fuse by exposure to IL-4 on non-treated tissue culture plastic, detached, and separated using cell sieves with 30- and 10-μm pore size. (B) Hemacolor-stained preparations before and after separation, viewed in bright field microscopy. (C) Comparison of expression of FcγRI, FcγRIIb, FcγRIII, and FcγRIV by western blotting of lysates of the separated cell populations. Cells were pretreated with LPS (10 ng/ml) for 24 hr before lysis. (D) Comparison of CD11b, CD11c, and CD18 expression by western blot. In (C) and (D), actin was used as loading control. Results are representative of greater than or equal to three independent experiments.

IL-4-induced BMM fusion on non-treated tissue culture plastic (Jay et al., 2007) allowed the detachment of macrophages and MGCs (Rosen and Gordon, 1987) and their separation on 30- and 10-μm pore size sieves (Figure 4A) into the two pure cell populations as seen in bright field microscopy (Figure 4B). This method enabled direct western blot comparisons of the expression of all four murine IgG receptors (FcγR) and complement receptors 3 (CR3) (CD11b/CD18) and 4 (CR4) (CD11c/CD18) on MGCs and M2 macrophages they derive from. Importantly, these cell populations were from the same culture and thus grown under the same conditions. Consistent with the reduced uptake of IgG-opsonized RBCs in MGCs (Figure 3C), there was reduced FcγRIIb and FcγRIV expression in fused compared to unfused macrophages under inflammatory conditions (Figure 4C). FcγRI and FcγRIII levels were unchanged. Complement receptor CD11b was similarly expressed in both populations, but expression of both CD11c and CD18 was greater in MGCs (Figure 4D), suggesting a possible role for CR4 (CD11c/CD18) in the increased uptake of C3-opsonized RBCs (Figure 1D).

### FcγRIV Promotes Phagocytosis of IgG-Opsonized Targets

Blocking the different FcγR subtypes in phagocytosis using specific anti-FcγR antibodies showed that the uptake of IgG-opsonized RBCs by macrophages was largely dependent on FcγRIV (Figure S2A) whereas blockade of FcγRIIb/III had no significant effect (Figure S2B). Combined with the observed reduction in FcγRIV protein expression (Figure 4C), these results indicate that reduced IgG-dependent phagocytosis by MGCs under inflammatory conditions (Figure 3C) reflects their reduced FcγRIV expression.

### CD11c/CD18 Expression Increases in Alternatively Activated Macrophages and Substitutes for CD11b/CD18 Integrin Adhesion

**Figure 5:**
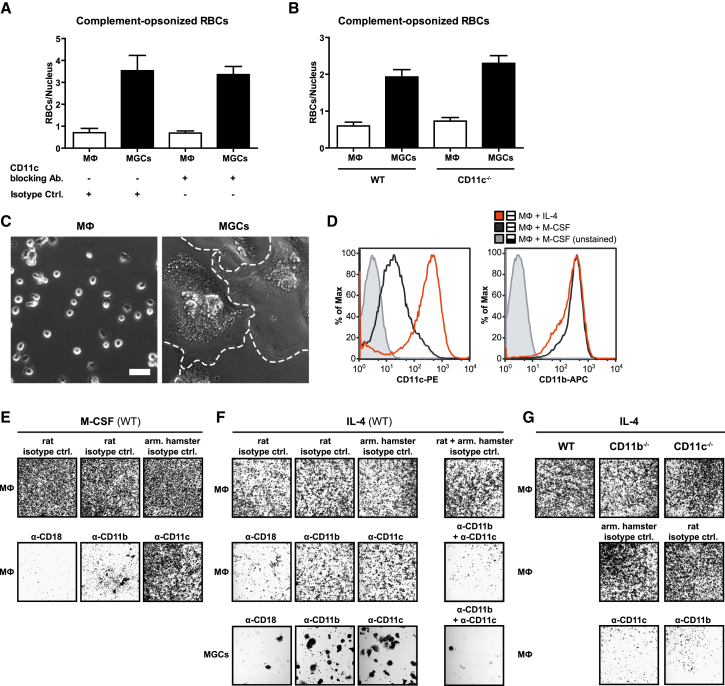
Alternative Macrophage Activation Increases CR4 Expression, which Can Substitute for CR3 in Integrin-Dependent Adhesion (A) Effect of CR4 (CD11c)-blocking antibodies on phagocytosis of complement-opsonized RBCs. MGC/macrophage cultures were preincubated with anti-CD11c (N418) or isotype control antibodies for 30 min on ice before addition of opsonized RBCs. (B) Phagocytosis of complement-opsonized RBCs in fused and unfused macrophages from CD11c-KO mice. (A) and (B) show means ± SEM, n = 25, and statistical comparison with isotype (A) or WT (B) controls. (C) Separated macrophages and MGCs (dashed lines) were plated on non-treated plastic for readhesion. After 3 hr at 37°C, non-adherent cells were washed off and images acquired by phase contrast microscopy. The scale bar represents 50 μm. (D) Three-day-old BMMs were cultured for further 3 days with M-CSF or with IL-4 instead on tissue-culture-treated plastic (non-fusogenic), immunostained with anti-CD11c-PE or anti-CD11b-APC antibodies, and analyzed by flow cytometry. (E–G) Cultures of WT (E and F) or knockout (G) cells were incubated with M-CSF or IL-4 for 3 days. Macrophages and MGC were separated and plated onto non-treated plastic for re-adhesion in the presence or absence of blocking antibodies, CD18 (GAME-46), CD11b (5C6), and CD11c (N-418) or isotype controls. After 3 hr, cells were washed and stained with crystal violet and results viewed by bright field microscopy. All results are representative of greater than or equal to three independent experiments. See also Figure S2.

**Figure 6:**
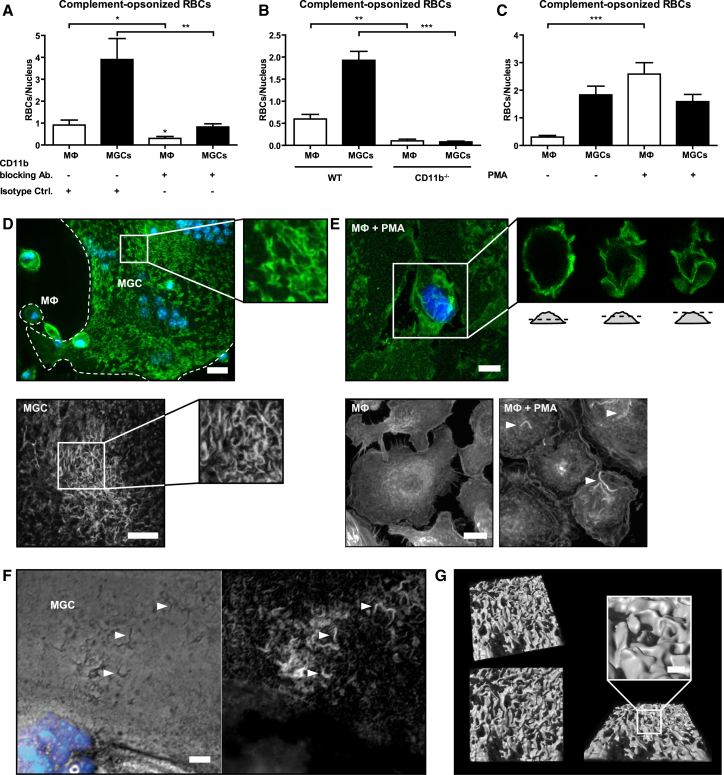
Activated CR3 and Extensive Membrane Ruffling Enable Increased Uptake of C3-Opsonized RBCs and Large Particles by MGCs (A) MGC/macrophage cultures were preincubated with CR3-blocking (anti-CD11b; 5C6) or isotype control antibodies (on ice; 30 min) before RBC addition. (B) Phagocytosis of complement-opsonized RBCs by CD11b-deficient macrophages/MGCs. (C) Internalized complement-opsonized RBCs in the absence/presence of PMA (150 ng/ml) after 1 hr of phagocytosis, normalized to the nucleus count. Results are shown as mean ± SEM, n = 25, and are representative of greater than or equal to three independent experiments. ^∗^p < 0.05; ^∗∗^p < 0.01; ^∗∗∗^p < 0.001; two-tailed Student’s t test. (D) (Top) Macrophages/MGCs (dashed lines) were stained with anti-CD11b, Alexa-488-labeled secondary antibodies (green), and nuclei counterstained with Hoechst (blue). The scale bar represents 20 μm. (Bottom) MGC stained with ConA-Alexa-488 (white; detail) is shown. Confocal images are shown as merged z stack. The scale bar represents 10 μm. (E) (Top) Macrophages treated with PMA (150 ng/ml) for 1 hr were stained as described in (D) (top). Confocal optical sections are depicted in detail. The scale bar represents 5 μm. (Bottom) Macrophages treated without/with PMA (150 ng/ml) for 1 hr were stained for polymerized actin with phalloidin-TRITC (white). Confocal images are shown as merged z stack. Arrows indicate membrane ruffles. The scale bar represents 10 μm. (F) MGCs were stained with phalloidin-TRITC (white) and Hoechst (blue) and images taken by fluorescence microscopy. Arrows indicate actin-containing membrane ruffles. The scale bar represents 10 μm. (G) 3D surface projection of a detailed section from (D) (bottom) generated by Imaris software. The scale bar represents 1 μm. See also Movies S3 and S4.

Surprisingly, although CR4 expression was found to be increased in MGCs (Figure 4D), there was no reduction in phagocytosis after treatment of MGCs with CD11c-blocking antibody (Figure 5A) or in MGCs derived from CD11c-KO mice (Figure 5B). Unrelated to its role in phagocytosis of complement-opsonized particles, complement receptor CR4 (CD11c/CD18) is an integrin involved in cellular adhesion. Indeed, consistent with the increased expression of CD11c/CD18 by MGCs, there was extensive cell spreading of IL-4-induced MGCs immediately after plating on non-treated plastic, a surface where cell adhesion is integrin dependent (Rosen and Gordon, 1987). Unfused M2 macrophages from the same culture retained a rounded shape typical of poorly adherent cells for the first few hours (Figure 5C). Expression of CD11c/CD18 protein was strongly upregulated by IL-4 stimulation, compared to macrophages kept in the presence of M-CSF as the crucial growth/survival factor, whereas expression of CD11b/CD18 was not affected (Figure 5D). Consistent with these protein findings, blocking antibodies to CD11b and CD18 inhibited adhesion of M-CSF macrophages (Figure 5E), as previously reported (Rosen and Gordon, 1987), but neither anti-CD11b nor anti-CD11c alone affected adhesion of MGCs or their M2 macrophage precursors (Figure 5F). Only combined CD11b and CD11c blockade or blockade of their common CD18 partner abolished cell adhesion in the presence of IL-4. Furthermore, M2 macrophages derived from either CD11b- or CD11c-knockout mice exhibited normal cell adhesion, but antibody blockade of the respective complementary integrin abolished adhesion (Figure 5G). Evidently and in contrast to M-CSF macrophages, CD11c/CD18 and CD11b/CD18 can substitute for each other in cell adhesion of IL-4-induced M2 macrophages and their MGC products, and the strongly increased CD11c/CD18 expression of M2 macrophages and MGCs (Figures 4D and 6D) allows for CD11c/CD18-mediated integrin adhesion, shifted away from the purely CD11b/CD18-mediated integrin adhesion observed for M-CSF macrophages. The enhanced spreading of MGCs relative to their M2 macrophage precursors (Figure 5C) appears connected to their elevated expression of CD11c/CD18 whereas the enhanced phagocytosis of complement-opsonized targets by MGCs is independent of CD11c.

### Increased Uptake of Large and C3-Opsonized Targets by MGCs Is Enabled by Activated CR3 and Extensive Membrane Ruffling

In contrast to CR4, blockade of complement receptor CR3 completely abolished the increased uptake of C3-opsonized RBCs by MGCs (Figure 6A), and such uptake was also absent in MGCs derived from BMMs of CD11b-knockout mice (Figure 6B). Despite the strict dependence of the enhanced capacity on CR3 for complement-mediated phagocytosis in MGCs, CR3 protein levels were not increased in MGCs (Figure 4D). However, CR3 is an integrin (Itgam/Itgb2) subject to bi-directional (inside-out and outside-in) signaling. PMA, which induces CR3 activation through inside-out signaling (Caron et al., 2000), markedly increased phagocytosis of C3-opsonized targets by unfused M2 macrophages but remarkably had no effect on their phagocytosis by MGCs (Figure 6C). Apparently CR3 on MGCs is in a pre-activated state and does not require further external stimuli to mediate maximally efficient phagocytosis.

Efficient phagocytosis via CR3 on PMA-activated macrophages has previously been shown to be facilitated by extensive plasma membrane ruffles (Patel and Harrison, 2008). The CR3 molecules of unstimulated MGCs, identified by confocal microscopy, were indeed located on just such membrane ruffles (Figure 6D), which closely resembled those on PMA-treated macrophages (Figure 6E). Ruffles, bearing abundant CR3, both on PMA-activated macrophages and MGCs, were rich in polymerized actin, and could be even visualized by light microscopy without staining (Figure 6F). Furthermore, 3D surface projection of confocal microscopy z stacks clearly showed the three-dimensional protrusion of ruffles from the surface of unstimulated MGCs (Figure 6G; Movies S3 and S4). Importantly, membrane ruffles provide a scaffold to which pre-activated CR3 localizes for efficient complement-mediated phagocytosis (Patel and Harrison, 2008) and also supply the excess cellular membrane necessary to cover and engulf large particles during phagocytosis (Cannon and Swanson, 1992). The extensive membrane ruffling present on MGCs in their natural unstimulated state was absent in surrounding unfused M2 macrophages, illustrating the unique and distinctive phenotype of MGCs that allows them to efficiently engulf large, complement-opsonized materials.

### MGC Formation Accompanies Phagocytosis of Complement-Opsonized AA Amyloid Deposits In Vivo

**Figure 7:**
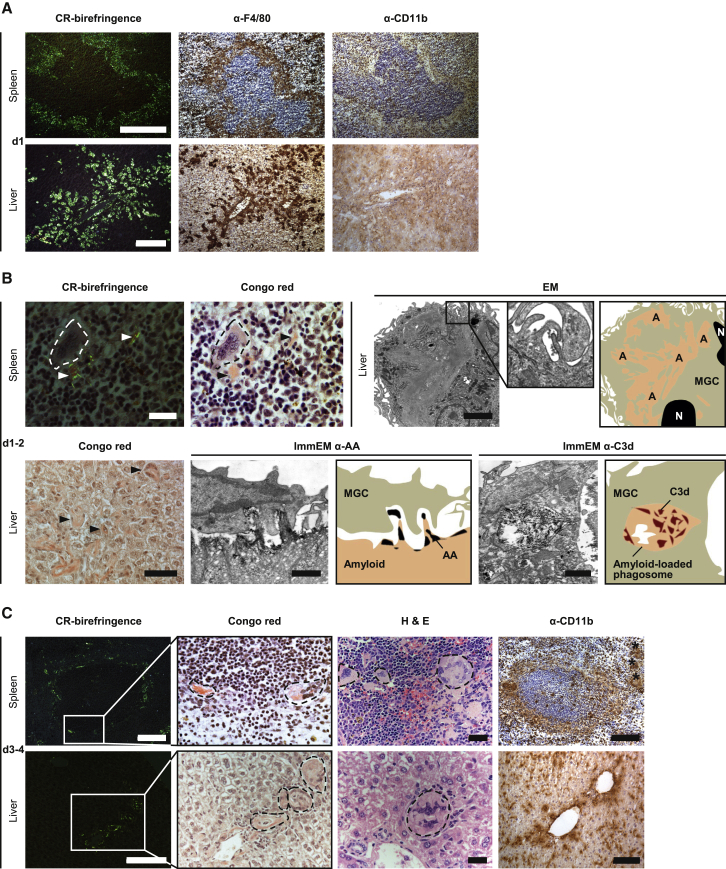
Complement Activation on Amyloid Deposits In Vivo Triggers Their Elimination by MGCs Representative histology and IHC of spleen and liver from AA amyloidotic mice that received a single dose of complement fixing anti-SAP antibodies and were sacrificed at intervals thereafter. (A) Within 24 hr of treatment, amyloid deposits (CR-birefringence) in the splenic perifollicular marginal zone and hepatic portal tracts were surrounded and robustly infiltrated by F4/80+ macrophages (α-F4/80). The scale bars represent 100 μm. (B) At days 1 or 2, the macrophages fused to form MGCs (spleen, dashed lines; scale bar represents 20 μm) that aligned closely to the extracellular amyloid (arrows). Amyloid (arrows) could also be seen inside some MGCs (liver, Congo-red; scale bar represents 50 μm). Transmission electron microscopy revealed the presence of extensive membrane ruffling on amyloid-laden MGCs (scale bar represents 2 μm; enlargement highlights membrane ruffling). Immunoelectron micrographs showed MGCs actively ingesting complement-opsonized AA amyloid (ImmEM α-AA) and phagosomes containing internalized C3d-positive amyloid fibrils inside MGCs (ImmEM α-C3d); scale bars represent 1 μm. A, amyloid; N, nucleus. Accompanying cartoons highlight the peripheral contours and relevant features of adjacent TEM images. (C) By days 3 or 4, extracellular amyloid was much reduced and fragmented (CR-birefringence; scale bars represent 100 μm). In parallel, MGCs (dashed lines) were now larger and their cytoplasm was filled with amyloid (Congo-red: congophilic intracellular amyloid; arbitrary bright field enlargement of boxed area from CR-birefringence; H&E: eosinophilic intracellular amyloid; scale bars represent 20 μm). They also strongly expressed CR3 (α-CD11b; scale bars represent 100 μm). Asterisks, amyloid-laden MGCs distant from original areas of amyloid deposition. Scale bars on the first LHS image of each panel in (A)–(C) apply also to adjacent images unless indicated otherwise. See also Figure S3.

Systemic amyloid A (AA) amyloidosis induced in mice by chronic inflammation closely resembles human AA amyloidosis. After histochemical staining with Congo red, the amyloid deposits, located in the splenic marginal zone and in hepatic periportal regions, exhibit pathognomonic bright green birefringence when viewed in intense cross-polarized light (Figure 7A). When these murine amyloid deposits were loaded with human serum amyloid P component (SAP) in vivo, administration of a single dose of complement-activating IgG anti-human SAP antibody triggered massive infiltration of F4/80-positive macrophages within 24 hr (Bodin et al., 2010; Figure 7A). In contrast, cellular infiltration was conspicuously and characteristically absent from the deposits of control amyloidotic mice receiving either no treatment or an irrelevant IgG antibody (Bodin et al., 2010). The infiltrating cells in anti-SAP-antibody-treated mice were initially only weakly positive for CD11b (Figure 7A) and the phagosome-lysosome marker CD68 (not shown), but within 1 or 2 days, there was extensive fusion to form MGCs with strong CD68 immunoreactivity (Bodin et al., 2010), and the majority of these were closely associated with the amyloid substance (Figure 7B). Indeed, amyloid was present within the cytoplasm of some cells (Figure 7B), and immunoelectron microscopy showed MGCs actively ingesting amyloid (Figure 7B). Moreover, extensive membrane ruffling, as seen in vitro (Figure 6), was present on MGCs that formed in vivo in relation to the amyloid deposits (Figure 7B). As expected, the MGCs were immunoreactive for F4/80, confirming their macrophage origin (Figure S3). Amyloid fibrils within phagosomes were positive for C3d (Figure 7B), confirming complement activation and deposition on the amyloid target, consistent with our previous demonstration in C1q- or C3-deficient mice that antibody-mediated elimination of amyloid was complement dependent (Bodin et al., 2010). By 3 or 4 days after anti-SAP treatment, there was a dramatic increase in macrophage fusion and in the size of the MGCs, which were surrounding and fragmenting the amyloid. Overall amyloid load was markedly reduced, and the residual deposits showed only dull green birefringence (Figure 7C). The majority of MGCs appeared completely filled with intracellular amyloid (Figure 7C), which was within multiple phagosomes and immunostained for the AA fibril protein (Bodin et al., 2010). We previously reported strong immunoreactivity of MGCs at this stage for CD68 (Bodin et al., 2010), the marker of phagolysosome fusion, and here, we observed an additional strong immunoreactivity for CD11b, peaking at this time point (Figure 7C). Some MGCs were no longer located adjacent to the original areas of amyloid deposition, and few mononuclear macrophages were found here also. By day 14, almost no amyloid was detectable and normal splenic and hepatic architecture was restored (Bodin et al., 2010). Crucially, the whole process was clinically silent with no adverse clinical, hematological, or biochemical effects observed in any recipient of the treatment.

## Discussion

Since the first description of MGCs in 1868, there have been few attempts to investigate their specific properties or to clarify their function. Different in vitro macrophage fusion systems (Helming and Gordon, 2007, Jay et al., 2007, McNally and Anderson, 1995) and fusion effector molecules have been identified (Aguilar et al., 2013, Helming and Gordon, 2009). However, none of these molecules are unique to fusing macrophages so they cannot be used in knockout approaches to prevent MGC formation and to investigate MGC function in vivo. Here, we studied mixed M2-activated macrophage-MGC cultures in vitro, combined with a method we established to separate MGCs and unfused macrophages from the same culture, and thus directly link phagocytosis and phagocytic receptor expression by the two related cell types. We found that MGCs phagocytosed IgG-opsonized RBCs less avidly than unfused M2 macrophages, consistent with our finding of reduced FcγR expression in MGCs. In contrast, MGCs were remarkably more competent in the uptake of large particles and were specifically equipped for phagocytosis of complement-opsonized targets via activated CR3.

Activation of CR3 is a prerequisite for efficient ingestion of complement-opsonized particles (Bianco et al., 1975) and can be induced by PMA or via adhesion of macrophages to permissive surfaces (Wright et al., 1983). Interestingly, although Wright et al. (1983) reported SAP to be such a permissive surface coating in vitro, macrophages almost never adhere spontaneously to amyloid fibrils in vivo, despite all such fibrils being invariably coated with SAP. This observation is consistent with the marked anti-opsonic effect of SAP coating of bacteria (Noursadeghi et al., 2000). Distinct from phagocytosis, the adhesion process itself is also important for macrophage fusion (Helming and Gordon, 2007, McNally and Anderson, 2002), and CD44, an adhesion receptor for several extracellular matrix components, is among the receptors associated with MGC formation (Sterling et al., 1998). In vitro, macrophage binding of the CD44 ligand hyaluronan or treatment of the cells with anti-CD44 antibodies increases CR3 activation and enhances phagocytosis of complement-opsonized RBCs (Vachon et al., 2007). The increased activation of CR3 in MGCs reported here may thus reflect specialized adhesion processes associated with macrophage fusion.

In addition to pre-activated CR3, we identified extensive membrane ruffling on unstimulated MGCs as a critical structural feature required for their capacity to engulf large targets. Importantly, CR3 was located on these ruffles. Membrane ruffling is an induced, active process involving actin polymerization, and such extensive ruffles, capturing RBCs for phagocytosis, have previously been reported only on PMA-activated macrophages (Patel and Harrison, 2008). Ruffling provides an increased surface area for particle attachment via ligation to phagocytic receptors, and its induction is evidently closely related to fusion of macrophages into MGCs. The actin polymerization-associated small GTPase, Rac1, is required for both membrane ruffle formation in macrophages (Ridley et al., 1992) and for formation of MGCs (Gonzalo et al., 2010, Jay et al., 2007). Moreover, the membrane protrusions, which were necessary for cell-cell fusion (Shilagardi et al., 2013), were lacking on osteoclasts of severely osteopetrotic Rac1/Rac2 knockout mice (Croke et al., 2011). Taken together, these observations indicate that MGC ruffle formation may be a direct consequence of the macrophage fusion process.

Distinct from signaling pathways, ruffles and the excess plasma membrane they provide are physically necessary for the ingestion of large materials by macrophages (Cannon and Swanson, 1992), especially in cases where particle volume exceeds the cell volume (Champion and Mitragotri, 2006). The inherent membrane ruffling of MGCs is also evidently crucial for their capacity to phagocytose large particles and to engulf non-particulate targets of varying sizes. Cell fusion inevitably increases the surface area:volume ratio. For example, a smoothly spherical MGC derived from 50 spherical macrophages would have only 27% of the original total membrane area (Figure S4). The excess membrane generated on fusion thus becomes available as membrane ruffles, providing a scaffold for activated CR3 and enabling engulfment of large and complement-opsonized materials.

We show here that CR4, a major complement receptor on macrophages and MGCs, is not required for the phagocytosis of complement-opsonized targets. Nonetheless, functioning as the CD11c/CD18 integrin, CR4 can substitute for CR3 (CD11b/CD18) in mediating adhesion stimulated by IL-4. IL-4 also triggers formation of MGCs in culture in vitro and during the foreign body reaction in vivo (Kao et al., 1995). In such a T_H_2-dominated environment, MGCs may thus utilize CR4 for cell adhesion, freeing pre-activated CR3 for phagocytosis of complement-coated targets. Indeed, strongly upregulated CR4 expression may have a more-important role during the foreign body reaction than previously considered. Binding of plasma proteins (Kourtzelis et al., 2013), especially complement proteins (Tang et al., 1998), to surgical implants contributes substantially to their pro-inflammatory effects. Complement receptor-mediated cell adhesion to biomaterials may be particularly significant for foreign targets, such as metal or polymer implants, that do not have intrinsic ligands for phagocytic cell receptors. Importantly, these complement proteins are not necessarily plasma derived as macrophages and other myeloid cells can produce them locally (Feldmann and Pepys, 1974, Gadjeva et al., 2002, Kopf et al., 1998, Verschoor et al., 2001, Verschoor et al., 2003).

Macrophages and MGCs are also crucial for the clinically silent physiological clearance of autologous debris and for tissue remodeling after injury, such as the elimination of hematomas or the remodeling of bone, respectively. Furthermore, as previously reported, macrophage-derived MGCs play a key role in antibody-mediated clearance of amyloid in vivo in a humanized mouse model of systemic amyloidosis (Bodin et al., 2010). In serial histological, immunohistochemical, and electron microscopic studies over the days after a single dose of anti-SAP antibody, almost all ingestion and destruction of the amyloid was shown unequivocally to be conducted by the MGCs (Bodin et al., 2010). These unique cells are evidently specifically equipped for this important function. Indeed, the sequence of complement opsonization, rapidly followed by macrophage invasion and fusion to form MGCs with strong CR3 expression, which then engulf and destroy massive amyloid deposits, is entirely consistent with the present in vitro observations. We have thus described here key aspects of the cell biology underlying the role of MGCs in physiological removal from the tissues of large-scale, abnormal, autologous debris.

The ongoing first clinical trial of anti-SAP antibody therapy in patients with systemic amyloidosis has confirmed that this intervention causes major complement consumption and produces rapid, asymptomatic, and clinically beneficial regression of amyloid deposits (Richards et al., 2015), presumably by triggering macrophage infiltration and MGC formation in humans as it does in mice. In addition to clinical development, relationships between human MGC function and efficiency of amyloid clearance in vitro and in vivo are under investigation. More generally, our work encourages the potential therapeutic engagement of MGC-mediated clearance in other conditions caused by abnormal deposition of extracellular materials. Conversely, specific inhibition of macrophage fusion by interfering with a dominant T_H_2 environment, for instance via treatments involving clinically approved IL-4 and IL-13 antagonists, may reduce the pathological conditions associated with the foreign body reaction when MGCs are unable to degrade targets of extrinsic origin.

## Experimental Procedures

### Mice

Wild-type, *C3*- (Wessels et al., 1995), *Itgam*-(CD11b) (Melo et al., 2000), and *Itgax*-(CD11c) (Wu et al., 2009) knockout (KO) mice as well as human SAP transgenic mice (Iwanaga et al., 1989) were all on C57BL/6 background. C5-deficient mice were FVB/N that carries the *HC*^*0*^ locus. All mice were housed under specific pathogen-free conditions. All experiments complied with local veterinary laws and institutional guidelines.

### Phagocytosis Assay

For phagocytosis assays, fused macrophages on Permanox slides (Nunc) were washed with PBS, and 200 μl serum-free X-VIVO 10 medium (LONZA) was added to the wells. In experiments involving blocking antibodies or inhibitors, these were added at this step. After brief incubation at 37°C (humidified; 5% CO_2_), the slides were cooled on ice, particles were added, and binding by macrophages was allowed for 30 min. Slides were then returned to 37°C to allow phagocytosis to proceed for defined time periods. Phagocytosis was stopped by placing the slides back on ice, and unbound particles were removed by washing with PBS. Particles not ingested by the cells were stained by incubation on ice for 20 min with primary antibodies specific for the different opsonins being tested: IgG or C3 on RBCs and OVA or IgG on beads. The method for inside-outside discrimination of phagocytosis targets was based on a previously published protocol (Criss and Seifert, 2006). Briefly, fluorescently labeled antibodies with selective specificity for the phagocytosis targets (RBC or beads) were added to the adherent cell cultures without prior cell-fixation or -permeabilization steps to ensure selective access of the antibodies to external, non-phagocytosed particles only. The procedure thus enables externally attached (fluorescently labeled) targets to be accurately distinguished from ingested (non-fluorescently labeled) particles. After washing with PBS, the cells were fixed for 10 min with 4% w/v paraformaldehyde (PFA) or ice-cold methanol for RBCs, washed again with PBS, and then stained with the appropriate fluoresceinated secondary antibodies (15 min; on ice). Internalized 0.5-μm beads were visualized after permeabilizing the cells with 0.1% v/v Triton X-100 in PBS for 5 min at RT, followed by a second round of particle-specific primary antibodies and differently fluorochrome-labeled secondary antibodies. Nuclei were counterstained with Hoechst 33258 and slides viewed by fluorescence microscopy (Leica DMRBE). Representative images were captured with an AxioCam MRm digital camera using Axiovision software (Zeiss).

### Separation of Mononucleated and Multinucleated Fused Macrophages

Macrophage fusion was induced on non-treated polystyrene 6-well plates (Greiner Bio-One). The cells were then detached using 10 mM EDTA in PBS and passed sequentially through first a 30-μm and then a 10-μm pore size sieve (Partec). The final flowthrough was a pure population of unfused mononucleated macrophages. The MGCs retained in the 30-μm sieve were flushed out with alpha-MEM medium containing 10% v/v FCS, and both cell populations were then resuspended in complete alpha-MEM containing IL-4 and cultured overnight (37°C; humidified; 5% CO_2_).

### Amyloid Studies

Amyloid studies were as previously reported (Bodin et al., 2010) and detailed in the Supplemental Experimental Procedures.

### Statistics

Bar diagrams were constructed using GraphPad Prism 5. Significance of differences between groups was sought by unpaired two-tailed Student’s t tests (GraphPad Prism 5).

## Author Contributions

L.H., R.M., and A.V. designed the in vitro macrophage and MGC experiments; R.M. and J.R. performed them; and R.M., J.R., L.H., A.V., and F.O.M. analyzed the data. S.G. and F.O.M. discussed the results. M.B.P. conceived, directed, and analyzed in vivo experiments with amyloidotic mice; G.A.T. designed, performed, and interpreted the histological and immunocytochemical studies of ex vivo amyloidotic tissues; and A.L. performed and interpreted the electron microscopy. L.H., R.M., A.V., G.A.T., and M.B.P. co-wrote the paper with comments from S.G. and F.O.M.

## Conflicts of Interest

M.B.P. is the inventor on patents covering SAP as a therapeutic target in amyloid-associated diseases which are owned by Pentraxin Therapeutics, Ltd. He owns shares in this company, which has licensed some of the intellectual property on SAP targeting to GlaxoSmithKline.

## Supplemental Information

Document S1. Supplemental Experimental Procedures and Figures S1–S4

Movie S1. 3D Visualization of Non-ingested 45-μm Bead, Related to Figure 2 MGCs and macrophages were incubated with 45 μm latex beads for 24 hrs. 3D visualization of z-stack confocal images used for Figure S1A (red: external beads; grey: cellular membrane). Movie was created using volume rendering of Imaris software with 24 frames per second display rate.

Movie S2. 3D Visualization of MGC-Ingested 45-μm Bead, Related to Figure 2 MGCs and macrophages were incubated with 45 μm latex beads for 24 hrs. 3D visualization of z-stack confocal images used for Figure S1B (grey: cellular membrane). Movie was created using volume rendering of Imaris software with 24 frames per second display rate.

Movie S3. MGC Membrane Ruffles Visualized by Confocal Microscopy, Related to Figure 6 Consecutive images of z-stack shown as merged image in Figure 6D (lower panel). Movie was created using ImageJ software with 10 frames per second display rate.

Movie S4. 3D Visualization of MGC Membrane Ruffles, Related to Figure 6 3D visualization of MGC membrane ruffles as shown in Figure 6G. 3D projection was created using surface rendering of Imaris software with 24 frames per second display rate.

Document S2. Article plus Supplemental Information

## References

[bib1] Aguilar P.S., Baylies M.K., Fleissner A., Helming L., Inoue N., Podbilewicz B., Wang H., Wong M. (2013). Genetic basis of cell-cell fusion mechanisms. Trends Genet..

[bib2] Anderson J.M., Rodriguez A., Chang D.T. (2008). Foreign body reaction to biomaterials. Semin. Immunol..

[bib3] Bianco C., Griffin F.M., Silverstein S.C. (1975). Studies of the macrophage complement receptor. Alteration of receptor function upon macrophage activation. J. Exp. Med..

[bib4] Bodin K., Ellmerich S., Kahan M.C., Tennent G.A., Loesch A., Gilbertson J.A., Hutchinson W.L., Mangione P.P., Gallimore J.R., Millar D.J. (2010). Antibodies to human serum amyloid P component eliminate visceral amyloid deposits. Nature.

[bib5] Cannon G.J., Swanson J.A. (1992). The macrophage capacity for phagocytosis. J. Cell Sci..

[bib6] Caron E., Self A.J., Hall A. (2000). The GTPase Rap1 controls functional activation of macrophage integrin alphaMbeta2 by LPS and other inflammatory mediators. Curr. Biol..

[bib7] Chambers T.J. (1977). Studies on the phagocytic capacity of macrophage polykaryons. J. Pathol..

[bib8] Champion J.A., Mitragotri S. (2006). Role of target geometry in phagocytosis. Proc. Natl. Acad. Sci. USA.

[bib9] Criss A.K., Seifert H.S. (2006). Gonococci exit apically and basally from polarized epithelial cells and exhibit dynamic changes in type IV pili. Cell. Microbiol..

[bib10] Croke M., Ross F.P., Korhonen M., Williams D.A., Zou W., Teitelbaum S.L. (2011). Rac deletion in osteoclasts causes severe osteopetrosis. J. Cell Sci..

[bib11] Feldmann M., Pepys M.B. (1974). Role of C3 in in vitro lymphocyte cooperation. Nature.

[bib12] Gadjeva M., Verschoor A., Brockman M.A., Jezak H., Shen L.M., Knipe D.M., Carroll M.C. (2002). Macrophage-derived complement component C4 can restore humoral immunity in C4-deficient mice. J. Immunol..

[bib13] Gonzalo P., Guadamillas M.C., Hernández-Riquer M.V., Pollán A., Grande-García A., Bartolomé R.A., Vasanji A., Ambrogio C., Chiarle R., Teixidó J. (2010). MT1-MMP is required for myeloid cell fusion via regulation of Rac1 signaling. Dev. Cell.

[bib14] Helming L., Gordon S. (2007). Macrophage fusion induced by IL-4 alternative activation is a multistage process involving multiple target molecules. Eur. J. Immunol..

[bib15] Helming L., Gordon S. (2009). Molecular mediators of macrophage fusion. Trends Cell Biol..

[bib16] Iwanaga T., Wakasugi S., Inomoto T., Uehira M., Ohnishi S., Nishiguchi S., Araki K., Uno M., Miyazaki J., Maeda S. (1989). Liver-specific and high-level expression of human serum amyloid P component gene in transgenic mice. Dev. Genet..

[bib17] Jay S.M., Skokos E., Laiwalla F., Krady M.M., Kyriakides T.R. (2007). Foreign body giant cell formation is preceded by lamellipodia formation and can be attenuated by inhibition of Rac1 activation. Am. J. Pathol..

[bib18] Kao W.J., McNally A.K., Hiltner A., Anderson J.M. (1995). Role for interleukin-4 in foreign-body giant cell formation on a poly(etherurethane urea) in vivo. J. Biomed. Mater. Res..

[bib19] Kopf M., Herren S., Wiles M.V., Pepys M.B., Kosco-Vilbois M.H. (1998). Interleukin 6 influences germinal center development and antibody production via a contribution of C3 complement component. J. Exp. Med..

[bib20] Kourtzelis I., Rafail S., DeAngelis R.A., Foukas P.G., Ricklin D., Lambris J.D. (2013). Inhibition of biomaterial-induced complement activation attenuates the inflammatory host response to implantation. FASEB J..

[bib21] Lai S., Zhou X. (2013). Inflammatory cells in tissues of gout patients and their correlations with comorbidities. Open Rheumatol. J..

[bib22] Langhans T. (1868). Über riesenzellen mit wandständigen kernen in tuberkeln und die fibröse form des tuberkels. Virchows Arch..

[bib23] Lay G., Poquet Y., Salek-Peyron P., Puissegur M.P., Botanch C., Bon H., Levillain F., Duteyrat J.L., Emile J.F., Altare F. (2007). Langhans giant cells from M. tuberculosis-induced human granulomas cannot mediate mycobacterial uptake. J. Pathol..

[bib24] McNally A.K., Anderson J.M. (1995). Interleukin-4 induces foreign body giant cells from human monocytes/macrophages. Differential lymphokine regulation of macrophage fusion leads to morphological variants of multinucleated giant cells. Am. J. Pathol..

[bib25] McNally A.K., Anderson J.M. (2002). Beta1 and beta2 integrins mediate adhesion during macrophage fusion and multinucleated foreign body giant cell formation. Am. J. Pathol..

[bib26] Melo M.D., Catchpole I.R., Haggar G., Stokes R.W. (2000). Utilization of CD11b knockout mice to characterize the role of complement receptor 3 (CR3, CD11b/CD18) in the growth of Mycobacterium tuberculosis in macrophages. Cell. Immunol..

[bib27] Michl J., Unkeless J.C., Pieczonka M.M., Silverstein S.C. (1983). Modulation of Fc receptors of mononuclear phagocytes by immobilized antigen-antibody complexes. Quantitative analysis of the relationship between ligand number and Fc receptor response. J. Exp. Med..

[bib28] Moreno J.L., Mikhailenko I., Tondravi M.M., Keegan A.D. (2007). IL-4 promotes the formation of multinucleated giant cells from macrophage precursors by a STAT6-dependent, homotypic mechanism: contribution of E-cadherin. J. Leukoc. Biol..

[bib29] Nakanishi-Matsui M., Yano S., Matsumoto N., Futai M. (2012). Lipopolysaccharide induces multinuclear cell from RAW264.7 line with increased phagocytosis activity. Biochem. Biophys. Res. Commun..

[bib30] Noursadeghi M., Bickerstaff M.C., Gallimore J.R., Herbert J., Cohen J., Pepys M.B. (2000). Role of serum amyloid P component in bacterial infection: protection of the host or protection of the pathogen. Proc. Natl. Acad. Sci. USA.

[bib31] Patel P.C., Harrison R.E. (2008). Membrane ruffles capture C3bi-opsonized particles in activated macrophages. Mol. Biol. Cell.

[bib32] Prokop S., Heppner F.L., Goebel H.H., Stenzel W. (2011). M2 polarized macrophages and giant cells contribute to myofibrosis in neuromuscular sarcoidosis. Am. J. Pathol..

[bib33] Richards D.B., Cookson L.M., Berges A.C., Barton S.V., Lane T., Ritter J.M., Fontana M., Moon J.C., Pinzani M., Gillmore J.D. (2015). Therapeutic clearance of amyloid by antibodies to serum amyloid P component. N. Engl. J. Med..

[bib34] Ridley A.J., Paterson H.F., Johnston C.L., Diekmann D., Hall A. (1992). The small GTP-binding protein rac regulates growth factor-induced membrane ruffling. Cell.

[bib35] Rosen H., Gordon S. (1987). Monoclonal antibody to the murine type 3 complement receptor inhibits adhesion of myelomonocytic cells in vitro and inflammatory cell recruitment in vivo. J. Exp. Med..

[bib36] Samokhin A.O., Wilson S., Nho B., Lizame M.L., Musenden O.E., Brömme D. (2010). Cholate-containing high-fat diet induces the formation of multinucleated giant cells in atherosclerotic plaques of apolipoprotein E-/- mice. Arterioscler. Thromb. Vasc. Biol..

[bib37] Schlesinger L., Musson R.A., Johnston R.B. (1984). Functional and biochemical studies of multinucleated giant cells derived from the culture of human monocytes. J. Exp. Med..

[bib38] Shilagardi K., Li S., Luo F., Marikar F., Duan R., Jin P., Kim J.H., Murnen K., Chen E.H. (2013). Actin-propelled invasive membrane protrusions promote fusogenic protein engagement during cell-cell fusion. Science.

[bib39] Sterling H., Saginario C., Vignery A. (1998). CD44 occupancy prevents macrophage multinucleation. J. Cell Biol..

[bib40] Tang L., Liu L., Elwing H.B. (1998). Complement activation and inflammation triggered by model biomaterial surfaces. J. Biomed. Mater. Res..

[bib41] Vachon E., Martin R., Kwok V., Cherepanov V., Chow C.W., Doerschuk C.M., Plumb J., Grinstein S., Downey G.P. (2007). CD44-mediated phagocytosis induces inside-out activation of complement receptor-3 in murine macrophages. Blood.

[bib42] Verschoor A., Brockman M.A., Knipe D.M., Carroll M.C. (2001). Cutting edge: myeloid complement C3 enhances the humoral response to peripheral viral infection. J. Immunol..

[bib43] Verschoor A., Brockman M.A., Gadjeva M., Knipe D.M., Carroll M.C. (2003). Myeloid C3 determines induction of humoral responses to peripheral herpes simplex virus infection. J. Immunol..

[bib44] Wessels M.R., Butko P., Ma M., Warren H.B., Lage A.L., Carroll M.C. (1995). Studies of group B streptococcal infection in mice deficient in complement component C3 or C4 demonstrate an essential role for complement in both innate and acquired immunity. Proc. Natl. Acad. Sci. USA.

[bib45] Wright S.D., Craigmyle L.S., Silverstein S.C. (1983). Fibronectin and serum amyloid P component stimulate C3b- and C3bi-mediated phagocytosis in cultured human monocytes. J. Exp. Med..

[bib46] Wu H., Gower R.M., Wang H., Perrard X.Y., Ma R., Bullard D.C., Burns A.R., Paul A., Smith C.W., Simon S.I., Ballantyne C.M. (2009). Functional role of CD11c+ monocytes in atherogenesis associated with hypercholesterolemia. Circulation.

