# Supplementary material for: Multinucleated Giant Cells Are Specialized for Complement-Mediated Phagocytosis and Large Target Destruction
Source: Cell Rep. 2015 Nov 25;13(9):1937–48. doi: 10.1016/j.celrep.2015.10.065 (PMC4675895; doi:10.1016/j.celrep.2015.10.065)
Supplement: Document S1. Supplemental Experimental Procedures and Figures S1–S4 [file mmc1.pdf]

Cell Reports

Supplemental Information

Multinucleated Giant Cells Are Specialized for Complement-Mediated Phagocytosis and Large Target Destruction

Ronny Milde, Julia Ritter, Glenys A. Tennent, Andrzej Loesch, Fernando O. Martinez, Siamon Gordon, Mark B. Pepys, Admar Verschoor, and Laura Helming

SUPPLEMENTAL DATA
Figure S1

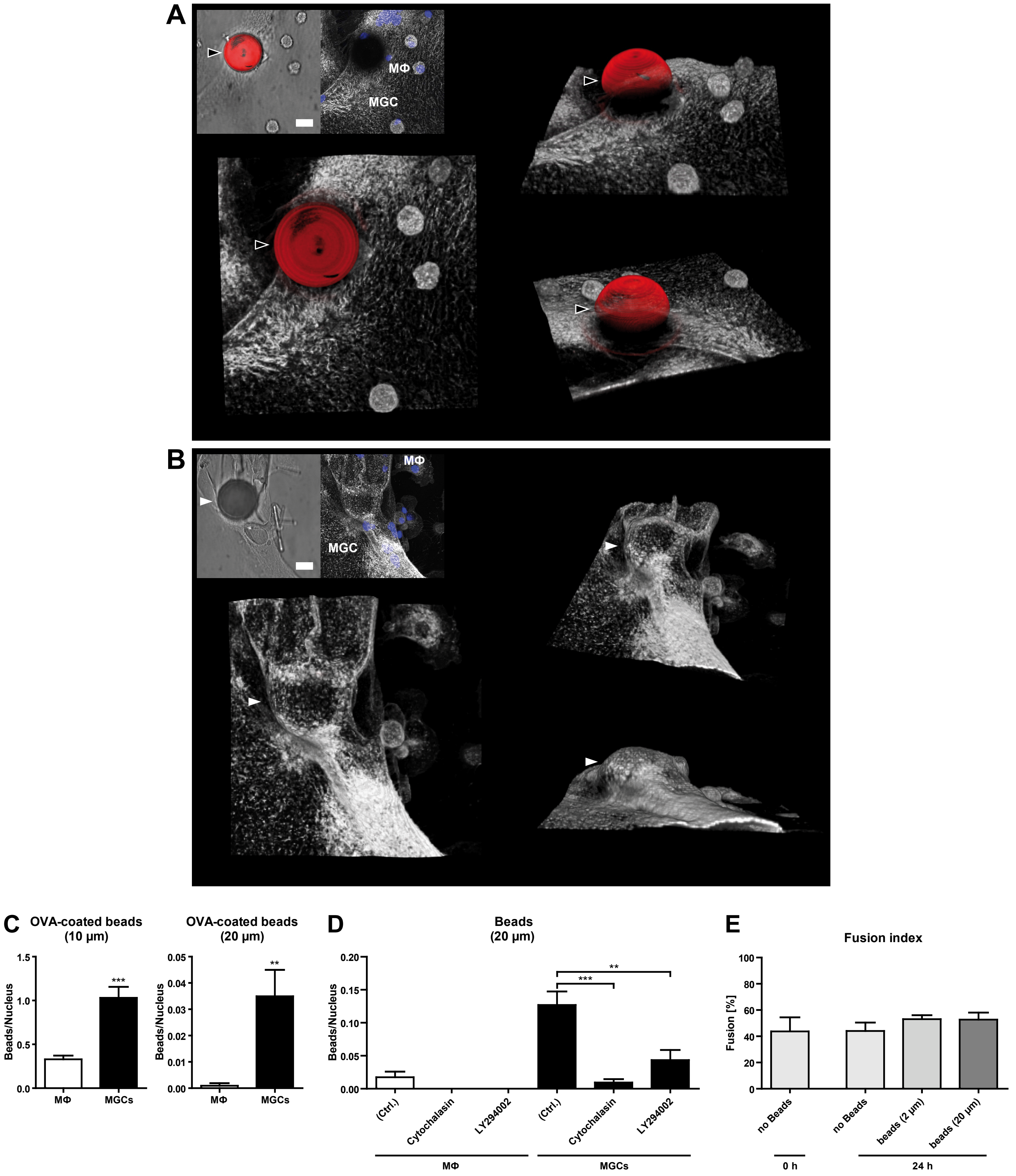

Figure S2

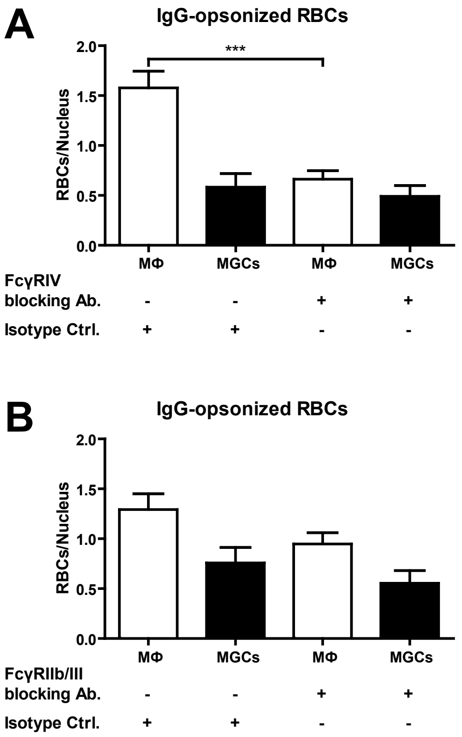
A
IgG-opsonized RBCs
RBCs/Nucleus
2.0
1.5
1.0
0.5
0.0
***
MΦ
MGCs
MΦ
MGCs
FcγRIV
blocking Ab.
-
-
+
+
Isotype Ctrl.
+
+
-
-
B
IgG-opsonized RBCs
RBCs/Nucleus
2.0
1.5
1.0
0.5
0.0
MΦ
MGCs
MΦ
MGCs
FcγRIIb/III
blocking Ab.
-
-
+
+
Isotype Ctrl.
+
+
-
-

Figure S3

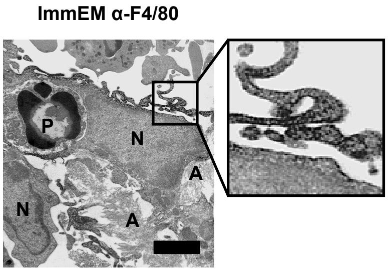

Figure S4

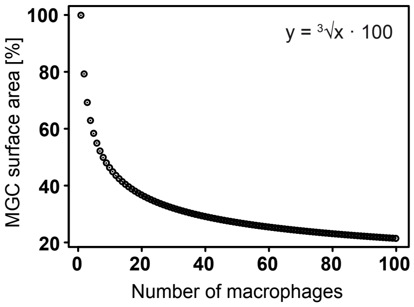
y = ³√x · 100
MGC surface area [%]
100
80
60
40
20
0
20
40
60
80
100
Number of macrophages

Figure S1. Large particle phagocytosis is independent of particle coating, follows normal macrophage phagocytosis mechanisms and is not based on macrophage fusion around particles; Related to Figure 2

(A, B) Detection of internalized 45 µm IgG-coated latex beads. External beads were immunostained with anti-rabbit DyLight549 antibodies (red), nuclei counterstained with Hoechst (blue) and membrane stained with Concanavalin A (ConA)-Alexa488 (grey). Arrows indicate non-ingested (black) or ingested (white) particles. Scale bar: 20 µm. Raw images, acquired by confocal microscopy, were volume rendered with Imaris software for 3D visualization.

(C) Quantification of phagocytosis of 10 and 20 µm latex beads coated with ovalbumin (OVA). External beads were excluded by immunostaining with anti-OVA antibodies.

(D) Effect of inhibitors on the phagocytosis of 20 µm latex beads by unfused macrophages and MGCs, normalized to the number of nuclei. Cytochalasin (1 µM, Sigma Aldrich) and LY294002 (50 µM, Sigma Aldrich) were present during the 24 hrs phagocytosis period.

(E) Quantification of macrophage fusion before and after addition of IgG-coated latex beads (2 and 20 µm diameter) for 24 hrs. Fusion index: percentage of giant cell nuclei related to the total number of nuclei.

Shown are means ± SEM, n=25 (A and B). Results are representative of ≥3 independent experiments. Statistical analyses were performed comparing to macrophages (A), control (B) or "no Beads 0 h" (C). **P<0.01, ***P<0.001, 2-tailed Student’s t-test. See also Movies S1 and S2.

Figure S2. FcγRIV is responsible for the phagocytosis of IgG-opsonized RBCs; Related to Figure 5

MGC/macrophage cultures were preincubated with (A) anti-FcγRIV (9E9), (B) anti-FcγRIIb/III (2.4G2) or isotype control antibodies for 30 min on ice before addition of the opsonized RBCs. Cells were pretreated with LPS (10 ng/ml) for 24 hrs (absent during phagocytosis assay). Results are shown as mean ± SEM, n=25 and are representative of ≥3 independent experiments. Statistically significant differences between antibody and isotype controls: ***P<0.001, 2-tailed Student's t-test.

Figure S3. MGCs associated with complement-opsonized amyloid deposits have F4/80-positive membrane ruffles *in vivo*; Related to Figure 7

Immunoelectron microscopy for F4/80 confirmed that the ruffled cells associated with *ex vivo* AA amyloid deposits were of macrophage origin (ImmEM α-F4/80, scale bar: 2 µm, enlargement highlights membrane ruffling). A, amyloid; N, nucleus; P, phagosome.

Figure S4. Mathematical model for the increase in excess membrane upon macrophage fusion; Related to Discussion

For the mathematical model shown here, cells were considered as spheres. With increasing numbers of macrophages that fuse to form one MGC (x-axis), the proportion of membrane area to cover that MGC relative to the original macrophage membrane area declines (y-axis). The underlying equation is shown in the top right corner. The graph was generated using R software.

Movie S1. 3D visualization of non-ingested 45 µm bead; Related to Figure 2

MGCs and macrophages were incubated with 45 µm latex beads for 24 hrs. 3D visualization of z-stack confocal images used for Figure S1A (red: external beads; grey: cellular membrane). Movie was created using volume rendering of Imaris software with 24 frames per second display rate.

Movie S2. 3D visualization of MGC-ingested 45 µm bead; Related to Figure 2

MGCs and macrophages were incubated with 45 µm latex beads for 24 hrs. 3D visualization of z-stack confocal images used for Figure S1B (grey: cellular membrane). Movie was created using volume rendering of Imaris software with 24 frames per second display rate.

Movie S3. MGC membrane ruffles visualized by confocal microscopy; Related to Figure 6

Consecutive images of z-stack shown as merged image in Figure 6D (lower panel). Movie was created using ImageJ software with 10 frames per second display rate.

Movie S4. 3D visualization of MGC membrane ruffles; Related to Figure 6

3D visualization of MGC membrane ruffles as shown in Figure 6G. 3D projection was created using surface rendering of Imaris software with 24 frames per second display rate.

SUPPLEMENTAL EXPERIMENTAL PROCEDURES

Antibodies

Anti-chicken egg ovalbumin (OVA-14) and IgG from rabbit serum were obtained from Sigma Aldrich. Anti-C3b/iC3b/C3c (3/26) antibodies were from Hycultec, anti-rabbit IgG DyLight549 (Cat# 111-505-003) and anti-rabbit IgG Alexa488 (Cat# 111-545-144) from Dianova, anti-rat IgG Alexa488 (Cat# A11006) and anti-mouse IgG Alexa488 (Cat# A11029) from Invitrogen. Anti-CD11b (5C6), anti-CD11c (N418) and anti-FcγR (2.4G2) were purified from hybridoma supernatants using a HiTrap MabSelect column (GE Healthcare). Anti-CD11b (M-19) and anti-FcγRI (N-19) used in Western blot analyses were obtained from Santa Cruz; anti-FcγRIII (EPR4333) was from Abcam, anti-CD11c (435421) from R&D and anti-Actin (C4) from Merck Millipore. Anti-FcγRIV (9E9) was a kind gift from Falk Nimmerjahn and anti-CD18 (pAb) kindly provided by Melanie Laschinger. Anti-CD11b-APC (M1/70) and anti-CD11c-PE (N418) used in flow cytometry were from Pharminogen and Biolegend, respectively.

Primary mouse macrophages and IL-4-induced macrophage fusion

BMMs were obtained by flushing femora and tibiae of at least 6 week old C57BL/6J male and female mice and culturing bone marrow cells in alpha-MEM medium (PAA) containing Penicillin/Streptomycin, 10% v/v fetal calf serum (FCS; Sigma Aldrich) (=complete alpha-MEM) and 15% v/v L929-supernatant as a source of M-CSF for 3 days on 6-well non-treated plastic plates (Greiner) in a humidified, 5% CO_2, 37°C incubator. Non-adherent cells were removed by washing with PBS and adherent macrophages detached using PBS containing 10 mM EDTA. Detached macrophages were plated on Permanox® 8-well chamber slides ($2x10^5$/well) in

complete alpha-MEM containing IL-4 as 1% supernatant derived from the mIL-4 cDNA-transfected X63Ag8–653 plasmacytoma transformant cell line (Karasuyama and Melchers, 1988) as described (Helming and Gordon, 2007), and incubated for 3 days (37°C, humidified, 5% CO_2) to induce macrophage fusion. For the separation of mono- and multinucleated cells, macrophages were differentiated for 3 days in M-CSF containing medium on non-treated plastic plates as described above. After removing non-adherent cells, complete alpha-MEM containing IL-4 was added directly to wells and the macrophages cultured (37°C, humidified, 5% CO_2) for 3 days to induce formation of MGCs.

Thioglycollate-elicited macrophages (ThioMs) were isolated by peritoneal lavage of at least 6 week old male and female C57BL/6J mice that had been injected with 3% v/v thioglycollate broth (Sigma Aldrich) in PBS 4 days previously. Macrophages were resuspended in complete alpha-MEM containing IL-4 and plated on Permanox® 8-well chamber slides ($1x10^5$/well) for 24 hrs (37°C, humidified, 5% CO_2) to induce macrophage fusion.

To visualize fusion, nuclei were stained with Hoechst 33258 (Sigma Aldrich) or Hemacolor® (Merck). Fusion index was determined by counting the nuclei in mono- and multinucleated cells in 3 different fields of view ($\Sigma n > 1600$) in fluorescence microscopy and by calculating the percentage of MGC-nuclei related to the total number of nuclei.

Differentiation of osteoclasts

After detaching 3 day old BMMs from the culture plate, cells were washed and resuspended in alpha-MEM (Sigma Aldrich). Cells ($3x10^4$ cells/well) were seeded in 96-well microplates (tissue culture treated, Becton Dickinson) and cultured in the

presence of 20 ng/ml M-CSF and 150 ng/ml RANKL (both from R&D Systems) at 37°C (humidified, 5% CO_2). Media were exchanged on days 2, 3, 4 and 5 and osteoclasts used on day 6 for the phagocytosis assay.

Opsonization of sheep RBCs with IgG or complement

For opsonization with IgG, 100 µl of sheep RBCs (1% v/v, Virion\Serion) were washed by centrifugation (2000 g, 1 min) with PBS and incubated with 1 µl of hemolytic amboceptor (rabbit anti-RBC antibodies, Virion\Serion) in a total volume of 200 µl for 30 min at 37°C with agitation. RBCs were then washed with PBS and resuspended in X-VIVO 10 medium (LONZA). To opsonize RBCs with complement, 100 µl of a 1% v/v RBC solution were diluted 1:2 in PBS and incubated with 1 µl of mouse anti-sheep-RBC specific IgM (Cedarlane) for 30 min at 37°C. After centrifugation (2000 g, 1 min), RBCs were resuspended in 100 µl FVB/N (C5-deficient) or C3-KO mouse serum. Following 1 h of incubation at 37°C, RBCs were washed again in PBS and resuspended in X-VIVO 10.

Coating polystyrene beads for phagocytosis

Carboxylated polystyrene beads (Polysciences) of different size (0.5-20 µm) were coated with rabbit IgG by carbodiimide binding. In brief, beads were washed and resuspended in 98.5 mM 2-(N-Morpholino)ethanesulfonic acid buffer (MES, pH 6.1) at 20 mg/ml and an equal volume of freshly prepared carbodiimide solution (10 mg/ml) was added. After incubation for 15 min with continuous mixing at RT, beads were washed and resuspended in borate buffer (50 mM H_3BO_3, 3.6 mM $Na_2B_4O_7$, pH 8.0) at 20 mg/ml. Then, 26.7 µl (for 0.5/2 µm beads) or 10 µl (for beads ≥6 µm) of rabbit IgG or 30 µl of ovalbumin (1 mg/ml, Sigma Aldrich) were added

followed by incubation for 2 hrs (RT) with continuous mixing. Beads were washed and resuspended in X-VIVO 10. Non-functionalized polystyrene beads of 45 µm diameter were coated by protein adsorption according to the manufacturer's instructions.

Staining of membrane actin and CD11b

For membrane staining, live cells plated on Permanox® 8-well chamber slides were incubated with 200 µl of 100 µg/ml concanavalin A (ConA) in PBS for 20 min at 37°C (humidified, 5% CO_2). For staining of polymerized actin, 5 µg/ml of phalloidin-TRITC was added after fixation with 4% w/v PFA and permeabilization with 0.1% v/v Triton X-100. For staining of CD11b, cells were fixed, incubated in blocking buffer (1% v/v normal goat serum, 0.1% v/v Tween 20) followed by incubation at RT with anti-CD11b (5C6) primary antibody (1 h) and secondary anti-rat Alexa488 (30 min). Nuclei were counterstained with Hoechst 33258 and slides mounted with Roti Mount Aqua (Carl Roth). Representative images were captured with an AxioCam MRm digital camera using Axiovision software (Zeiss).

3D visualization

3D visualization of images captured with a Leica TCS SP5 confocal microscope was performed by volume- or surface-rendering using Imaris version 7.6.5 (Bitplane).

Adhesion assay

Blocking antibodies against CD11b (clone 5C6), CD11c (clone N418), CD18 (clone GAME-46) or the corresponding isotype control antibodies, each in solution in alpha-MEM, were dispensed to individual wells of a non-treated polystyrene 96-well

plate (Greiner Bio-One). The different macrophage populations suspended in complete alpha-MEM were added to the antibody-containing wells ($1x10^5$ cells/well), incubated for 20 min on ice and then for 2 hrs at 37°C (humidified, 5% CO_2), before aspirating the fluid, washing the cells carefully with PBS and staining them with 1% w/v crystal violet for 10 min at RT.

Western blot

Adherent mono- and multinucleated cells were covered with ice-cold cell lysis buffer (50 mM Tris- HCl pH 7.5, 150 mM NaCl, 2.5 mM EDTA, 1% NP40, 0.5% $NaVO_4$, 0.1% NaF) containing Complete Protease Inhibitor Cocktail Tablets (Complete Mini, Roche Applied Science) and lysed for 30 min at 4°C. Total protein concentration of the lysates was determined using the bicinchoninic acid (BCA) assay (Pierce). Protein samples (2-10 µg) were applied to 6-14% SDS-PAGE gels, electrophoresed under reducing (CD11c, FcγRI, FcγRIII, CD18, Act) or non-reducing (CD11b, FcγRIIb/III, FcγRIV) conditions and transferred to nitrocellulose or PVDF (CD11b) membranes (0.45 µm pore size) by semi-dry blotting, using standard procedures. After incubation of membranes with relevant primary antibodies (overnight, 4°C) and corresponding HRP-conjugated secondary antibodies (1 h at RT), detection was performed using ECL Western Blotting Substrate (Pierce).

Flow cytometry

For surface expression analysis of integrins, macrophages were cultured (37°C, humidified, 5% CO_2) on non-fusogenic 6-well surface plates (tissue culture treated plastic, VWR). After washing with PBS, cells were scraped off with a pipette tip, briefly washed in a 96-well V-bottom microplate (non-tissue culture treated, Peske)

and resuspended in FACS blocking buffer (10% v/v normal goat serum, 1% w/v BSA, 0.1% v/v Tween 20 in PBS). Following 30 min incubation, cells were stained with integrin specific fluorochrome labeled antibodies in blocking buffer for 1 h on ice. After washing, cells were resuspended in 200 µl PBS and fixed by adding an equal volume of 4% w/v PFA. Cells were acquired on a CyAn ADP Lx (DakoCytomation) flow cytometer and data analyzed and visualized with FlowJo software.

Amyloid studies

Systemic AA amyloidosis was induced in a cohort of female human SAP transgenic C57BL/6 x 129/sv mice (wild-type SAP $^{+/+}$, F2) (Iwanaga et al., 1989), weighing 22-25 g and aged 10-12 weeks, by intravenous administration of amyloid enhancing factor followed by repeated subcutaneous injection of casein, as reported previously (Botto et al., 1997). Mice were then dosed with CPHPC, to deplete circulating SAP, followed by injection of either polyclonal IgG sheep anti-human SAP antibody or control sheep IgG (produced in-house), exactly as previously reported (Bodin et al., 2010). Nine groups, each of 4-5 mice, were killed at 1-4, 7, 10, 14, 21 and 25 days after a single i.p. injection of antibody. Separate pieces of each spleen and liver were either fixed in 10% v/v neutral buffered formalin and wax-embedded for haematoxylin and eosin (H & E) histology (2 µm sections), or unfixed, OCT-embedded and snap frozen for cryotomy and immunocytochemistry (Figure 7). Monoclonal rat anti-mouse F4/80 (clone A3-1; Abd Serotec) or anti-mouse CD11b (clone 5C6; AbD Serotec), and biotinylated affinity purified donkey anti-rat IgG secondary antibody (Cat# 712-065-153; Jackson ImmunoResearch), were used to identify canonical macrophages and CR3 expression respectively (Figure 7) by the avidin-biotin peroxidase method (VectaStain Elite ABC, Vector Labs) as follows.

Serial 6 µm cryosections were post-fixed in 2% w/v PFA (in Hepes-buffered saline with 2.7 mM $CaCl_2$; on ice, 10 min) and permeabilized (PBS-0.1% v/v Triton X-100; RT, 5 min), before quenching the endogenous peroxidase (10 mM glucose, 1.5 U/ml glucose oxidase, 1 mM NaN_3 in 0.1M $NaPO_4$ buffer pH 7.2; 37°C, 30 min), non-specific binding (20% v/v normal donkey serum in PBS-0.1% BSA, 0.01% Triton-X-100; RT, 30 min), and avidin-biotin (Avidin Biotin Blocking Kit, Vector Labs) activities. The sections were then incubated at 4°C overnight with optimal dilutions of primary antibodies, then washed in PBS before staining with the secondary antibody and avidin DH-biotinylated HRP complexes (both at RT, 30 min each). Peroxidase activity was visualized with metal enhanced diaminobenzidene (Thermo Fisher Scientific) and nuclei were counterstained with Mayer's haematoxylin (Pioneer Research Chemicals). After dehydration in graded ethanol baths to xylene, stained sections were mounted in DPX. Amyloid was identified in separate adjacent 6 µm cryosections, post-fixed as above, by its binding of Congo red under alkaline alcoholic staining conditions (Puchtler et al., 1962) to produce a characteristic pink-red color in bright light and vivid green birefringence in high-intensity cross-polarized light microscopy (Figure 7). Stained sections were examined with the DMR XA2 microscopy system controlled by QWIN Pro software (version 3.2), and representative images captured with a DFC 300FX digital camera (Leica Microsystems). Final images were prepared in Adobe PhotoShop.

Remaining portions of spleen and liver from representative treated and untreated amyloidotic mice were fixed in 4% w/v PFA-0.1% v/v glutaraldehyde in PBS and processed for either routine TEM (Figure 7B) or immunoelectron microscopy (ImmEM; Figures 7B and S3). For the latter, vibratome sections of the fixed tissues were processed for immunohistochemistry using the pre-embedding

ExtrAvidin-HRP technique. In brief, sections were quenched for endogenous peroxidase (hydrogen peroxide-methanol) and avidin-biotin (Avidin-Biotin Blocking kit, Vector Labs) activities, then incubated for 16 hrs at RT with optimal dilutions of polyclonal rabbit anti-mouse AA (produced in-house), rabbit anti-human/mouse C3d (Cat# A0063; Dako), or monoclonal rat anti-mouse F4/80 (clone A3-1; AbDSerotec). After subsequent incubation for 2.5 hrs at RT in affinity-purified biotinylated donkey anti-rabbit (Cat# 711-065-152; Jackson ImmunoResearch) or anti-rat (Cat# 712-065-153; Jackson ImmunoResearch) IgG and ExtrAvidin-HRP (Sigma Aldrich), immunoreactivity was visualized with diaminobenzidine (Sigma Aldrich). Stained sections were then fixed in osmium tetroxide, dehydrated in ethanol-propylene oxide and embedded in Araldite. Subsequently, ultrathin 80 nm sections were cut from flat embedded tissues (Vibratome sections) and contrasted with uranyl acetate and lead citrate, and examined using a Philips CM-120 transmission electron microscope. Representative images were acquired with an AMT 4000M T1 CL 2k side mount digital camera (Deben). Positive immunostaining appeared as a black, electron dense, precipitate that decorated the outer surface of the cellular plasma membrane (Figure S3, α-F4/80) or individual amyloid fibrils or bundles thereof (Figure 7B, α-AA, α-C3d). Uniformly negative controls confirmed immunospecificity of all immunostaining. The results shown are representative of independent experiments performed on tissue sections of at least 4 mice/time point.

SUPPLEMENTAL REFERENCES

Botto, M., Hawkins, P.N., Bickerstaff, M.C.M., Herbert, J., Bygrave, A.E., McBride, A., Hutchinson, W.L., Tennent, G.A., Walport, M.J., and Pepys, M.B. (1997). Amyloid deposition is delayed in mice with targeted deletion of the serum amyloid P component gene. Nature Med. *3*, 855-859.

Karasuyama, H., and Melchers, F. (1988). Establishment of mouse cell lines which constitutively secrete large quantities of interleukin 2, 3, 4 or 5, using modified cDNA expression vectors. Eur. J. Immunol. *18*, 97-104.

Puchtler, H., Sweat, F., and Levine, M. (1962). On the binding of Congo red by amyloid. J. Histochem. Cytochem. *10*, 355-364.
