# Supplementary figures and images for: Multinucleated Giant Cells Are Specialized for Complement-Mediated Phagocytosis and Large Target Destruction
Source: Cell Rep. 2015 Nov 25;13(9):1937–48. doi: 10.1016/j.celrep.2015.10.065 (PMC4675895; doi:10.1016/j.celrep.2015.10.065)

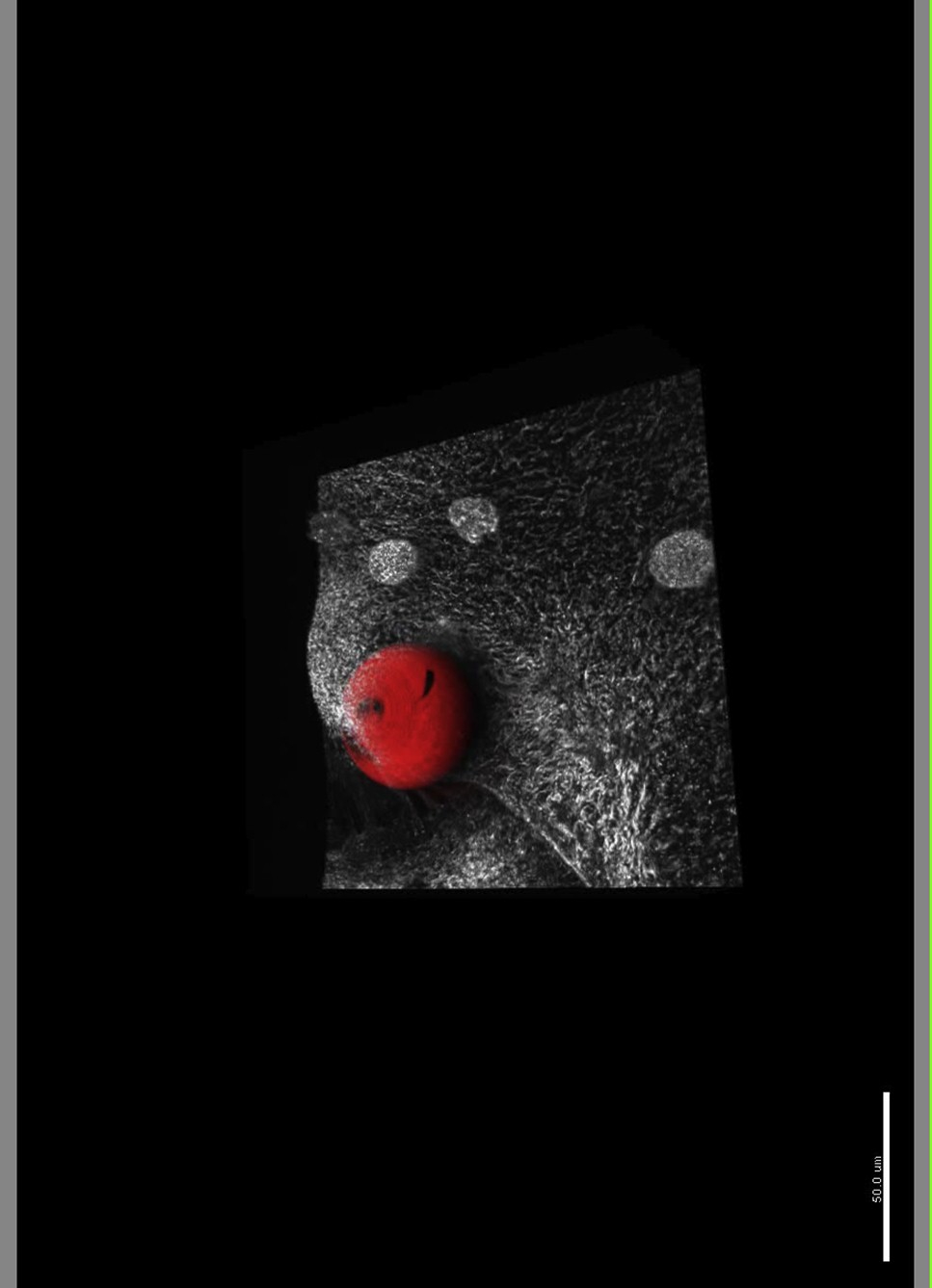

Supplement: Movie S1. 3D Visualization of Non-ingested 45-μm Bead, Related to Figure 2 — MGCs and macrophages were incubated with 45 μm latex beads for 24 hrs. 3D visualization of z-stack confocal images used for Figure S1A (red: external beads; grey: cellular membrane). Movie was created using volume rendering of Imaris software with 24 frames per second display rate. [file mmc2.jpg]

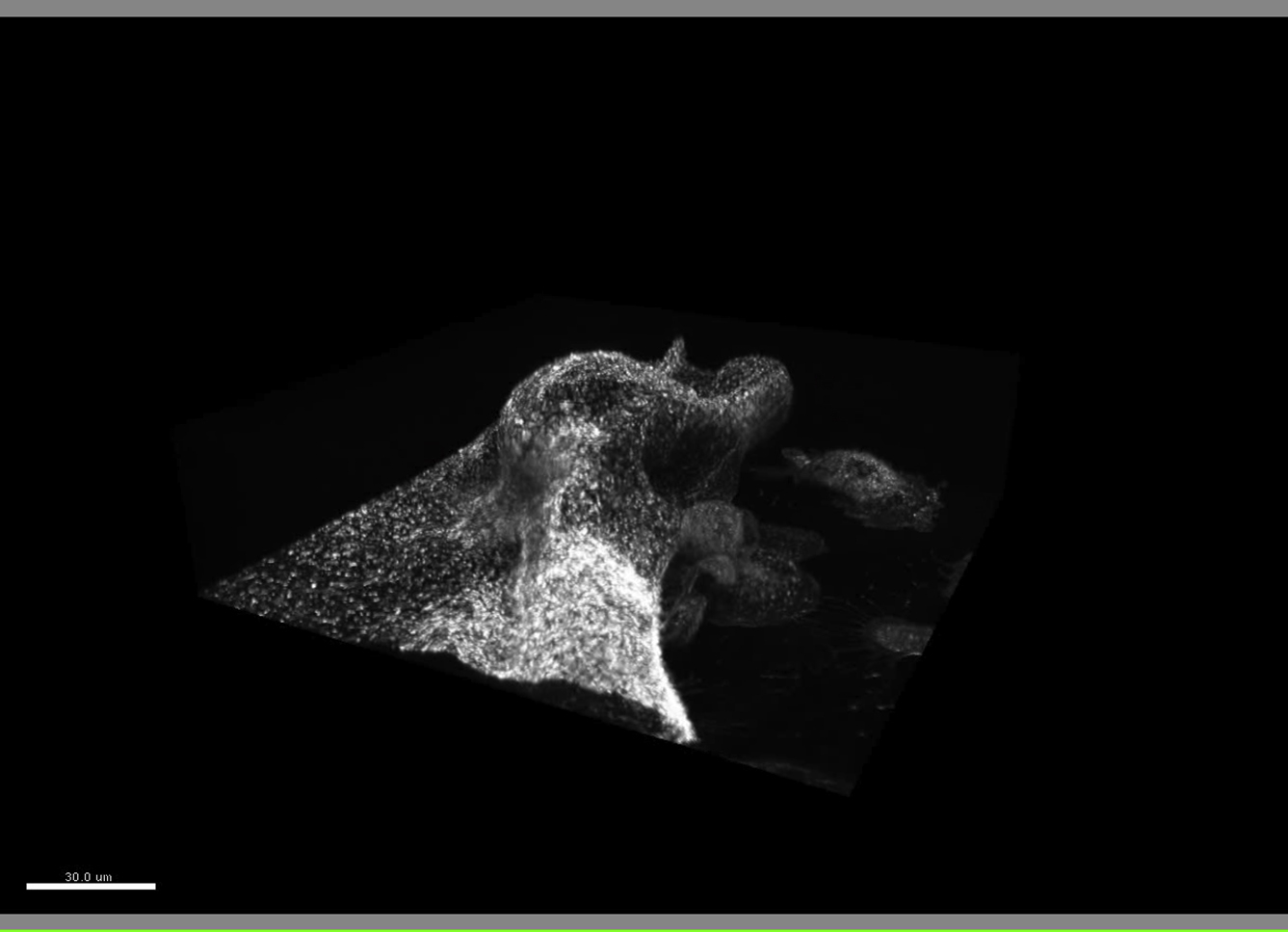

Supplement: Movie S2. 3D Visualization of MGC-Ingested 45-μm Bead, Related to Figure 2 — MGCs and macrophages were incubated with 45 μm latex beads for 24 hrs. 3D visualization of z-stack confocal images used for Figure S1B (grey: cellular membrane). Movie was created using volume rendering of Imaris software with 24 frames per second display rate. [file mmc3.jpg]

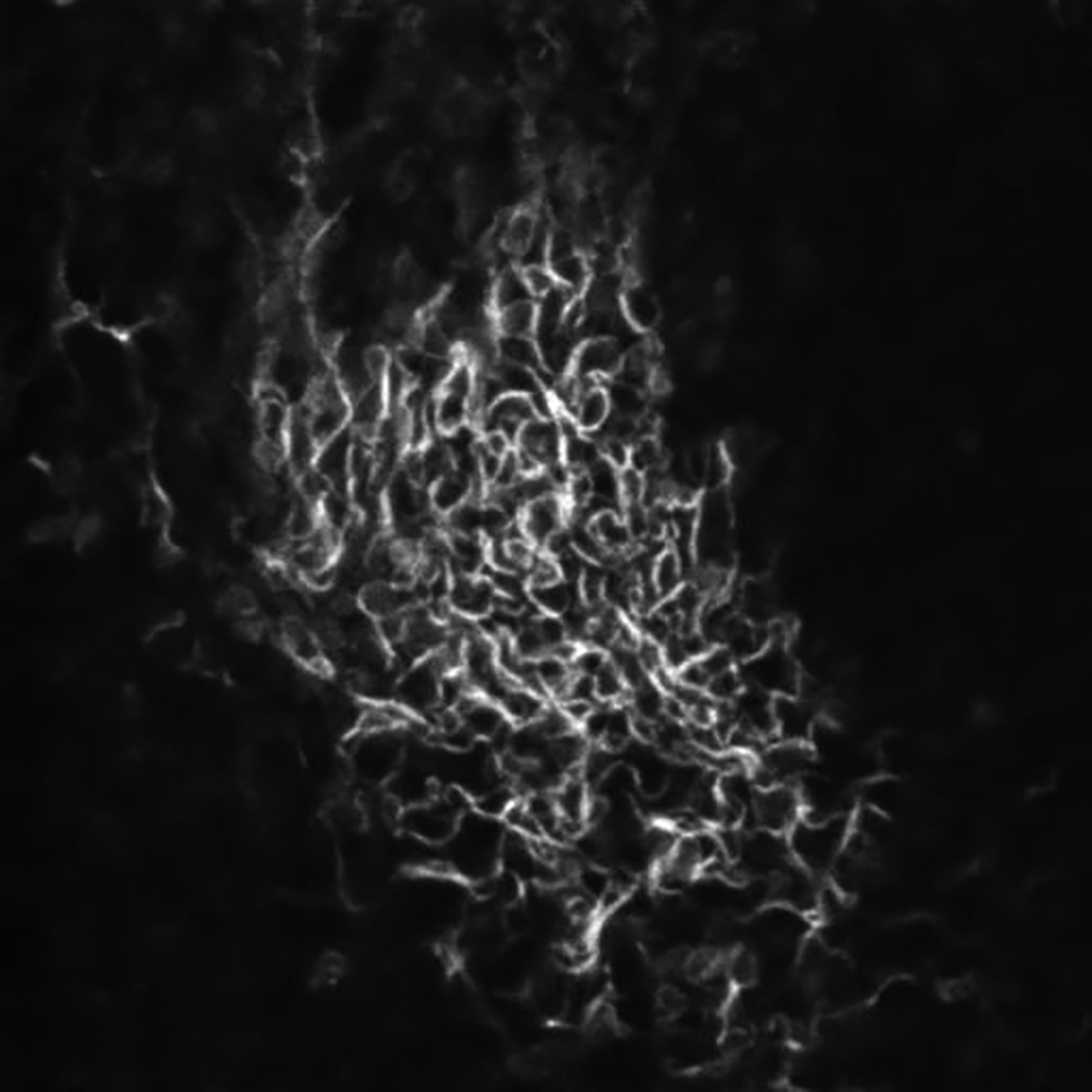

Supplement: Movie S3. MGC Membrane Ruffles Visualized by Confocal Microscopy, Related to Figure 6 — Consecutive images of z-stack shown as merged image in Figure 6D (lower panel). Movie was created using ImageJ software with 10 frames per second display rate. [file mmc4.jpg]

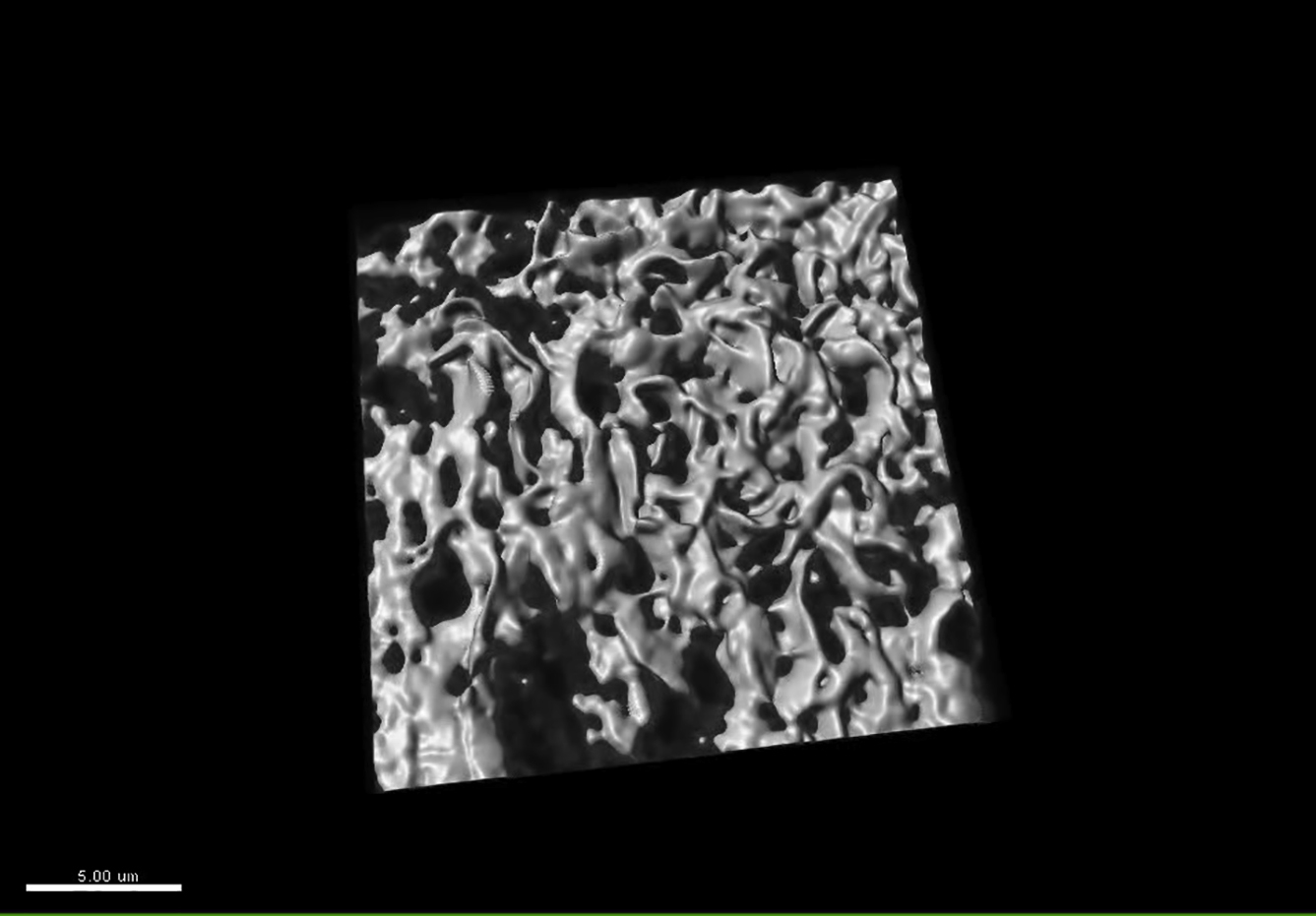

Supplement: Movie S4. 3D Visualization of MGC Membrane Ruffles, Related to Figure 6 — 3D visualization of MGC membrane ruffles as shown in Figure 6G. 3D projection was created using surface rendering of Imaris software with 24 frames per second display rate. [file mmc5.jpg]
